# Tuning SAS-6 architecture with monobodies impairs distinct steps of centriole assembly

**DOI:** 10.1038/s41467-021-23897-0

**Published:** 2021-06-21

**Authors:** Georgios N. Hatzopoulos, Tim Kükenshöner, Niccolò Banterle, Tatiana Favez, Isabelle Flückiger, Virginie Hamel, Santiago Andany, Georg E. Fantner, Oliver Hantschel, Pierre Gönczy

**Affiliations:** 1 Swiss Institute for Experimental Cancer Research (ISREC), School of Life Sciences, Swiss Federal Institute of Technology Lausanne (EPFL), Lausanne, Switzerland; 2 Laboratory for Bio- and Nano-Instrumentation, Swiss Federal Institute of Technology Lausanne (EPFL), Lausanne, Switzerland; 3 Present Address: Department of Plant Molecular Biology (DBMV), University of Lausanne, Lausanne, Switzerland; 4 Present Address: Department of Cell Biology, Sciences III, University of Geneva, Geneva, Switzerland; 5 Present Address: Institute of Physiological Chemistry, Faculty of Medicine, Philipps-University of Marburg, Marburg, Germany

**Keywords:** Cell biology, Cell division, Chemical biology, Structural biology, X-ray crystallography

## Abstract

Centrioles are evolutionarily conserved multi-protein organelles essential for forming cilia and centrosomes. Centriole biogenesis begins with self-assembly of SAS-6 proteins into 9-fold symmetrical ring polymers, which then stack into a cartwheel that scaffolds organelle formation. The importance of this architecture has been difficult to decipher notably because of the lack of precise tools to modulate the underlying assembly reaction. Here, we developed monobodies against *Chlamydomonas reinhardtii* SAS-6, characterizing three in detail with X-ray crystallography, atomic force microscopy and cryo-electron microscopy. This revealed distinct monobody-target interaction modes, as well as specific consequences on ring assembly and stacking. Of particular interest, monobody MB_CRS6_-15 induces a conformational change in CrSAS-6, resulting in the formation of a helix instead of a ring. Furthermore, we show that this alteration impairs centriole biogenesis in human cells. Overall, our findings identify monobodies as powerful molecular levers to alter the architecture of multi-protein complexes and tune centriole assembly.

## Abstract (web summary)

Centriole biogenesis begins with self-assembly of SAS-6 proteins into 9-fold symmetrical ring polymers, which then stack into a cartwheel that scaffolds organelle formation. Here, the authors develop monobodies against Chlamydomonas reinhardtii SAS-6 and use X-ray crystallography, atomic force microscopy and cryo-electron microscopy to reveal insights into ring assembly and stacking.

## Introduction

Centrioles are ninefold symmetrical cylindrical organelles conserved in structure and function from algae to men. Centrioles seed the formation of the ciliary axoneme across the eukaryotic tree of life and recruit the pericentriolar material of centrosomes in animal cells^1,2^. As a result, centrioles are fundamental for cell signaling and motility, as well as for cell polarity and division. Reflecting such fundamental roles, defects in centriole structure, number or function cause a range of human diseases^3–7^. Although the mechanisms governing centriole formation are increasingly well understood, how the striking architecture of the organelle relates to its function remains incompletely understood, including because of the lack of reagents to modulate the geometry of this multi-protein complex.

Most proliferating cells harbor two resident centrioles at the onset of the cell cycle (Supplementary Fig. 1a). In human cells, one procentriole emerges once per cell cycle from the surface of a torus surrounding the proximal part of each resident centriole^8–10^. Three proteins are most critical for the onset of procentriole assembly: Polo-like-kinase 4 (Plk4), STIL and HsSAS-6. In the current working model, Plk4 is present initially throughout the torus before focusing to a single location on its surface, notably owing to an autophosphorylation mechanism that targets the remainder of Plk4 for degradation, thus marking the unique position from which the procentriole emerges^8–10^.

Centrinone is a small molecule that has been developed as a potent and specific inhibitor of Plk4 activity^11^. Centrinone prevents procentriole formation in cultured human cells and has proven to be instrumental for unraveling many aspects of centriole biology^11–15^. Moreover, Centrinone may hold therapeutic potential for disease conditions with centriole number alterations, as well as in some specific cancer settings^16,17^. Despite these important advances and promises, Centrinone is not without limitation. First, Plk4 protein levels accumulate upon Centrinone treatment, owing to a lack of Plk4 autophosphorylation and thereby of Plk4 degradation, which typically translates into supernumerary centrioles upon drug release^11^. Second, Centrinone acts as a binary switch for centriole assembly and is difficult to use for modulating the assembly process in a more nuanced manner. Thus, the development of alternative means to tune centriole assembly holds important promise both as tool compounds and as novel therapeutic avenues.

One interesting alternative target in this regard is the evolutionarily conserved SAS-6 protein family^18–24^. In human cells, HsSAS-6 is essential for the onset of procentriole formation and accumulates on the torus where Plk4 has focused. SAS-6 proteins contain an N-terminal globular head domain followed by a long coiled-coil, and a C-terminal region predicted to be unstructured. SAS-6 proteins form homodimers in parallel and in register through a strong interaction between their coiled-coils^25–27^. Moreover, SAS-6 homodimers can form higher order oligomers through a weaker interaction between two head domains from neighboring homodimers, with a ~40° angle between the two coiled-coil pairs^25–27^. This can lead to the formation of a ninefold symmetrical ring polymer harboring 9 SAS-6 homodimers^25–27^. Such SAS-6 ring polymers resemble one layer of the cartwheel element that is observed by electron microscopy at the onset of procentriole formation, and which is thought to scaffold assembly of the emerging organelle^28–30^. Furthermore, SAS-6 ring polymers possess an intrinsic ability to stack on top of one another, resulting in vertical elements whose architecture resembles that of the entire cartwheel observed in the cellular context^31^.

Bioengineering approaches relying on the introduction of structure-guided mutations in SAS-6 protein have been pursued to alter centriole architecture^32^. However, such approaches necessitate complex genetic modifications. We sought to pursue an alternative approach that does not rely on such modifications, using monobodies instead. Monobodies are ~10 kDa synthetic binding proteins built using the tenth fibronectin type III (FN3) domain of human fibronectin as a molecular scaffold^33,34^. Monobodies with high affinity and selectivity to chosen targets can be developed by sequential phage and yeast display, sorting from large combinatorial libraries with diversified segments of this scaffold (Supplementary Fig. 1b). Over the past decade, monobodies have been developed to efficiently target and modulate signal transduction pathways, including the Bcr-Abl tyrosine kinase involved in leukemogenesis^35^, establishing these reagents as potent intracellular antagonists with utility in furthering mechanistic understanding of the underlying processes^36–39^. Moreover, monobodies can be potent modulators of enzymatic specificity^40^, as well as promising diagnostic and therapeutic tools. Here, we report the selection and characterization at the structural and functional levels of monobodies targeting and tuning the architecture of SAS-6 protein oligomers in the context of centriole assembly.

## Results

### Selection of monobodies against CrSAS-6

**Fig. 1:**
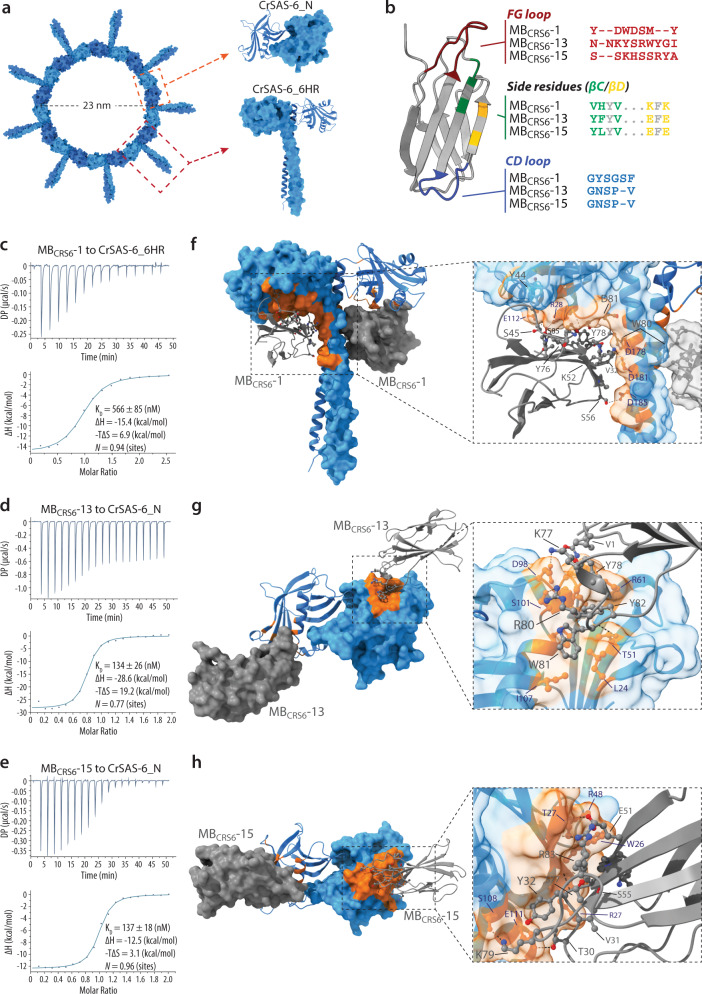
Development of monobodies against CrSAS-6. **a** CrSAS-6 homodimers (in blue) form ring polymers ~23 nm in diameter (left). Higher magnification views on the right show targets utilized for monobody selection: CrSAS-6_N (top) and CrSAS-6_6HR (bottom). **b** Monobody ribbon representation. The variable regions in the side-and-loop monobody library are colored: FG loop in red, side residues in connecting ßC/ßD strands in green and yellow, respectively, CD loop in blue. The amino acid sequences of the variable region for MB_CRS6_-1, MB_CRS6_-13 and MB_CRS6_-15 are shown on the right. **c**-**e** ITC profiles for the interaction between the targets CrSAS-6_6HR or CrSAS-6_N and the monobodies MB_CRS6_-1 (**c**), MB_CRS6_-13 (**d**), and MB_CRS6_-15 (**e**). **f**-**h** Structures of CrSAS-6_6HR (**f**) or CrSAS-6_N (**g**, **h**) in surface and ribbon representation (blue), highlighting in orange the residues interacting with MB_CRS6_-1 (**f**), MB_CRS6_-13 (**g**), and MB_CRS6_-15 (**h**), which are shown in gray in surface and ribbon representation, also in the higher magnifications on the right.

We set out to select monobodies recognizing *Chlamydomonas reinhardtii* CrSAS-6. We chose the SAS-6 protein from this species because it is amenable to cell free assays to probe both ring assembly and ring stacking, providing the potential to precisely test the consequences of selected monobodies. Moreover, crystal structures are available for the N-terminal globular domain of CrSAS-6 (termed CrSAS-6_N), as well as for a longer polypeptide containing also part of the coiled-coil domain (termed CrSAS-6_6HR to reflect the inclusion of six heptad repeats) (Fig. 1a, Supplementary Fig. 1c).

We sought to select monobodies from a combinatorial side-and-loop library (see Methods), in which the FG loop and the CD loop are randomized, as are several side residues in the connecting ßC/ßD strands (Fig. 1b)^41^. We prepared biotinylated CrSAS-6_6HR and CrSAS-6_N as targets for monobody selection (Supplementary Fig. 1d). Size exclusion chromatography established that both biotinylated targets were not aggregated (Supplementary Fig. 1e), while circular dichroism spectra demonstrated the presence of the expected secondary structure content in both cases (Supplementary Fig. 1f), together indicative of well-folded proteins suitable for the selection process. Biotinylated CrSAS-6_6HR and CrSAS-6_N were utilized to select interacting monobodies through successive sorting steps of phage and yeast display (Supplementary Fig. 1b) (Methods). Over forty monobody clones were identified initially, which comprised 14 unique sequences that were further characterized. Nine of these were selected against CrSAS-6_6HR (MB_CRS6_-1 through MB_CRS6_-9) and five against CrSAS-6_N (MB_CRS6_-11 through MB_CRS6_-15). Sequence analysis of the variable regions of the 14 monobodies revealed a large diversity of residues for each randomized segment, possibly suggestive of varied binding modes (Fig. 1b, Supplementary Fig. 2a). We recombinantly expressed and purified the majority of these monobodies (9/14) in high yield and determined the dissociation constant (K_D_) with their target using Isothermal Titration Calorimetry (ITC), finding K_D_s typically in the 100 nM range (Supplementary Fig. 2a, Supplementary Table 1). Overall, we conclude that we have identified a diverse set of monobodies recognizing CrSAS-6 with sub-micromolar affinities.

### Monobodies interact with different surfaces on their CrSAS-6 target

We report hereafter an in-depth characterization of three representative monobodies (see Methods for selection criteria): MB_CRS6_-1, which was selected against CrSAS-6_6HR (K_D_ ~566 nM, Fig. 1c), as well as MB_CRS6_-13 (K_D_ ~134 nM, Fig. 1d) and MB_CRS6_-15 (K_D_ ~137 nM, Fig. 1e), which were both selected against CrSAS-6_N. The three monobodies did not interact unspecifically with BSA (Supplementary Fig. 2b). We also investigated whether the three monobodies were specific to the target they were selected against, or else interacted also with the other CrSAS-6 protein target and the equivalents fragments of the human protein HsSAS-6. We found that MB_CRS6_-1 recognized CrSAS-6_6HR but not CrSAS-6_N, indicating that residues in the coiled-coil are essential for binding in this case (Supplementary Fig. 2b,; Supplementary Table 1). Moreover, MB_CRS6_-1 also interacted with the two equivalent fragments of HsSAS-6 at low but not at high concentration, presumably reflecting its low affinity (Supplementary Fig. 2b). We found also that MB_CRS6_-13 and MB_CRS6_-15 interacted not only with CrSAS-6_N, against which they were selected, but also CrSAS-6_6HR, demonstrating that the coiled-coil does not prevent binding (Supplementary Fig. 2b, c). MB_CRS6_-13 and MB_CRS6_-15 did not cross-react with HsSAS-6 fragments, underscoring their high specificity (Supplementary Fig. 2b).

In order to identify the binding epitope of the three monobodies to their respective target, we determined their X-ray co-crystal structure. MB_CRS6_-1 was crystallized with CrSAS-6_6HR harboring the mutation F145E to prevent the interaction between N-terminal domains that would result in a poly-disperse specimen hindering crystallization^25^. The co-crystal diffracted to 2.93 Å resolution and the structure was solved with molecular replacement and refined to R/R_free_ 24.4/29.1 (Fig. 1f, Supplementary Fig. 3a, 3d; Table 1). The final model in the asymmetric unit (ASU) consisted of three copies of the MB_CRS6_-1—CrSAS-6_6HR complex, arranged as homodimers that complete the biological units through symmetry related molecules. The interaction between MB_CRS6_-1 and CrSAS-6_6HR was similar in all three copies, extending over ~947 Å^2^. All variable regions of MB_CRS6_-1 contributed to the interaction through hydrogen bonds, a salt bridge, as well as several van der Waals and hydrophobic interactions (Fig. 1f, Supplementary Fig. 3a). To determine the impact of MB_CRS6_-1 binding on CrSAS-6_6HR, we compared the structure of the complex with that of CrSAS-6_6HR alone, which uncovered a conformational tilt of ~7° (Supplementary Fig. 3d). Although small, this tilt is expected to propagate upon higher order oligomerization of CrSAS-6 and potentially result in a ring polymer with a larger diameter (see below). Interestingly, despite the presence of the F145E mutations that prevents interaction between N-terminal domains^25^, CrSAS-6_6HR preserved this interaction in the crystal structure in the presence of MB_CRS6_-1, suggestive of a stabilizing effect.

**Table 1:** Data collection and refinement statistics.

	CrSAS-6_6HR-MB_CRS6_-1	CrSAS-6_N-MB_CRS6_-13	CrSAS-6_N-MB_CRS6_-15	CrSAS-6_6HR-MB_CRS6_-15
PDB ID	6ZZC	6ZZD	6ZZG	6ZZ8
Data collection				
Space group	C2	C2	C222_1_	P4_2_2_1_2
Cell dimensions				
a, b, c (Å)	153.4 123.2 73.1	107.9, 65.7, 90.4	164.5 347.2 94.9	257.2 257.2 125.3
α, β, γ (°)	90, 108, 90	90, 109, 90	90, 90, 90	90, 90, 90
Resolution range (Å)	4747.07-2.93 (3.11-2.93)	42.74-2.05 (2.13-2.05)	49.4-2.93 (3.07-2.93)	49.63 -3.73 (3.96-3.73)
R_merge_ (%)	8.1 (163.3)	8.6 (145.1)	27.1 (210.0)	22.2 (264.9)
Completeness (%)	97.5 (95.7)	98.7 (97.0)	99.0 (99.5)	99.8 (99.4)
Redundancy	2.1 (2.1)	6.9 (6.8)	4.5 (4.5)	7.9 (8.0)
I/σI	9.15 (0.7)	13.0 (1.3)	6.0 (0.8)	7.7 (0.85)
CC_1/2_ (%)	99.9 (74.3)	99.9 (64.5)	98.8 (86.5)	100.0 (59.1)
Refinement statistics				
R_work_ (%) (reflections)	24.4 (50608)	19.9 (68803)	22.1 (107315)	22.5 (78369)
R_free_ (%) (reflections)	29.1 (2652)	22.8 (3622)	25.3 (5671)	26.7 (4139)
Number of atoms				
Protein atoms	6937	3887	11201	14316
Ligands	37	0	20	0
Water	59	155	24	19
Average B factors (Å^2^)				
Protein atoms	125.1	73.01	83.5	227.5
Water	88.4	57.33	55.1	87.1
RMSD from ideal values				
Bonds / angles (Å/°)	0.005 / 0.9	0.01 / 1.2	0.003 / 0.8	0.003 / 0.7
Ramachandran plot				
Favored (%)	94.7	95.5	97.7	95.4
Disallowed (%)	0.46	0.41	0.43	0.39

Values in parentheses correspond to the high-resolution shell.

The X-ray co-crystal structure of MB_CRS6_-13 and CrSAS-6_N was determined at 2.05 Å resolution (Fig. 1g, Supplementary Fig. 3b, e; Table 1). The final model, refined to R/R_free_ 19.9/22.8, consisted of two copies of each molecule in the ASU, with the biological unit assembled through the symmetry related molecules that interacted with CrSAS-6-N. MB_CRS6_-13 binding to CrSAS-6_N is mediated mainly through residues of the FG loop, covering a surface area of ~642 Å^2^ (Fig. 1g, Supplementary Fig. 3b). MB_CRS6_-13 does not confer major changes to the overall structure of CrSAS-6_N, as indicated by a low RMSD of 0.857 Å compared to CrSAS-6_N alone (Supplementary Fig. 3e). Interestingly, however, we noted that MB_CRS6_-13 is positioned above and below the plane defined by CrSAS-6_N domains when present in the ring polymer (Supplementary Fig. 3e).

We also determined the X-ray crystal structure of MB_CRS6_-15 complexed with its target CrSAS-6_N to 2.93 Å resolution (Fig. 1h, Supplementary Fig. 3c, 3f; Table 1). There are six copies of the MB_CRS6_-15 – CrSAS-6_N complex in the ASU, each with a similar interaction interface of ~515 Å^2^, which is contributed by diversified residues of the FG loop and connecting ßC/ßD strands, further assisted by constant amino acids of the FN3 scaffold (Fig. 1h, Supplementary Fig. 3c). Similar to the crystal structure of CrSAS-6_N in its native form, the molecules are arranged in head-to-head dimers here as well, with small variations between them, likely due to crystal packing (Supplementary Fig. 3f).

Overall, we characterized structurally three monobodies that interact with distinct regions and have different structural consequences on their CrSAS-6 targets.

### AFM and cryo-EM assays uncover the impact of CrSAS-6 monobodies on ring assembly and stacking

**Fig. 2:**
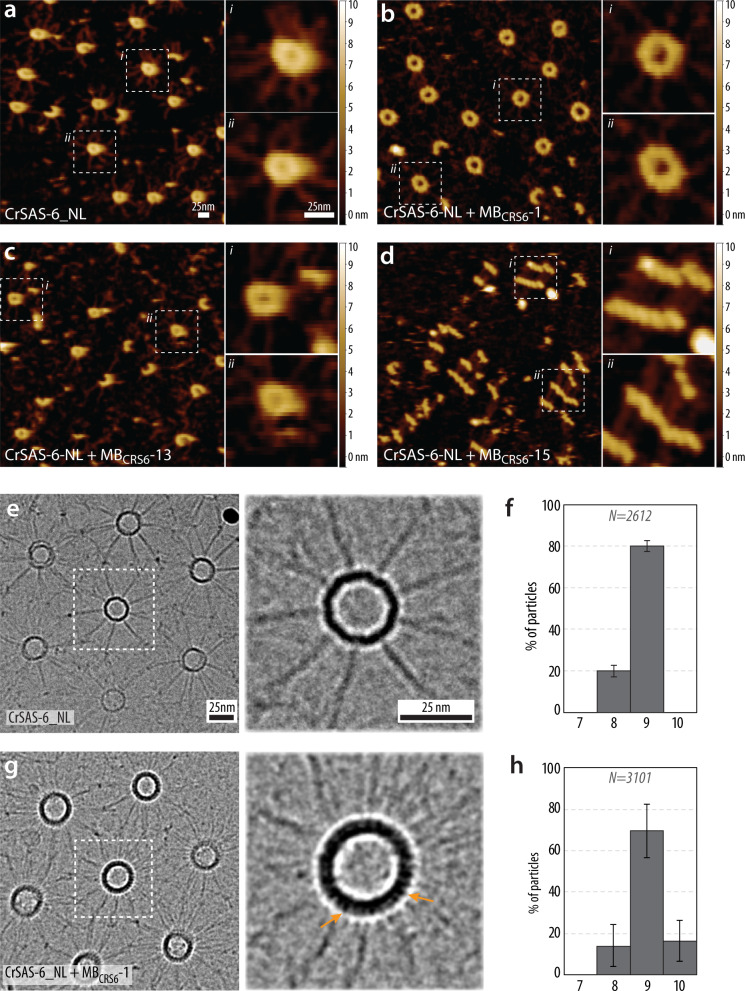
PORT-HS-AFM and cryo-EM uncover impact of monobodies on CrSAS-6 ring assembly and stacking. **a**-**d** PORT-HS-AFM equilibrium analysis of CrSAS-6_NL alone (**a**), or together with MB_CRS6_-1 (**b**), MB_CRS6_-13 (**c**), or MB_CRS6_-15 (**d**), with higher magnification views on the right showing in each case two representative examples of assemblies. Scale bars: 25 nm; z scale color bar, –0.5 to 10 nm. All measurements were performed at least twice, with at least 5 field of view imaged in each experiment. **e**-**h** Transmission cryo-EM of ring stacking assay^42^ and corresponding symmetry distributions based on particle classification of CrSAS-6_NL alone (**e**, **f**) or CrSAS-6_NL and a 50% molar excess of MB_CRS6_-1 (**g**, **h**). Note that spokes can be difficult to discern and be more numerous than 9, due to partially out of register stacking of SAS-6 ring polymers. The orange arrows point to protrusions observed on the outside of SAS-6 rings, corresponding to the density contributed by the monobody. Data represent mean ± SD. Particles numbers from at least 3 independent experiments: CrSAS-6_NL *N* = 2612, CrSAS-6_NL + MB_CRS6_-1 *N* = 3101.

We used two cell-free assays to test the impact of the three monobodies on the ability of CrSAS-6 to undergo ring assembly and ring stacking, respectively. We first report our findings using Photothermally actuated Off Resonance tapping High-Speed Atomic Force Microscopy (PORT-HS-AFM) to probe CrSAS-6 ring assembly^26^. In this assay, homodimers of CrSAS-6_NL, comprising the N-terminal head domain and the entire coiled-coil (Supplementary Fig. 1c), adsorb on the mica surface, where they self-assemble into ring polymers that can be monitored at equilibrium by PORT-HS-AFM (Fig. 2a). We incubated CrSAS-6_NL with each of the monobodies at a concentration that achieved at least 70% bound state (Methods). We found that the addition of MB_CRS6_-1 was of no apparent consequence on the ability of CrSAS-6_NL to form rings at equilibrium (Fig. 2b). MB_CRS6_-13 addition also enabled rings to be present (Fig. 2c), although they tended to be less frequent, perhaps reflecting a bona fide minor impact on the assembly. Alternatively, being positioned above and below the plane of the ring polymer (see Supplementary Fig. 3e), MB_CRS6_-13 might interfere with the ability of CrSAS-6 to adsorb properly on the mica surface. Although a slight increase in maximal ring height was observed in select rings upon addition of MB_CRS6_-1 or MB_CRS6_-13 (Supplementary Fig. 4a), the PORT-HS-AFM data set was too variable even with the CrSAS-6 control sample to reliably assess potential differences upon monobody addition (Supplementary Fig. 4b). Importantly, in addition, the impact of MB_CRS6_-15 as revealed by PORT-HS-AFM was striking: no rings were present at equilibrium, and instead linear assemblies were generated (Fig. 2d).

We also used a cryo-electron microscopy (cryo-EM) assay^42^ to test the impact of the three monobodies on SAS-6 ring stacking, a process that cannot be monitored using the PORT-HS-AFM assay conditions^26^. CrSAS-6_NL possesses an intrinsic ability to stack and form a lattice of interconnected cartwheel-like elements that can be analyzed by cryo-EM (Fig. 2e, Supplementary Fig. 4c, d)^42^. Measurements of ring diameter in such lattices indicated that ~80% of assemblies formed by CrSAS-6_NL exhibited ninefold radial symmetry, with the remaining ~20% being eightfold symmetric (Fig. 2f, Supplementary Fig. 4h), in line with previous observations^42^. We found that addition of MB_CRS6_-1 in stoichiometric amount with CrSAS-6_NL also yielded an interconnected lattice of cartwheel-like elements (Fig. 2g). MB_CRS6_-1 could be detected in such lattices on the outside of the CrSAS-6_NL ring (Fig. 2g, inset, arrows), as anticipated from the co-crystal structure (see Fig. 1f). Furthermore, measurements of ring diameter revealed a slight shift of symmetries, now including some tenfold symmetrical assemblies (Fig. 2h, Supplementary Fig. 4g–i). Together, these findings are compatible with the suggestion from the co-crystal structure that MB_CRS6_-1 binding might result in the formation of larger ring polymers. Using the cryo-EM stacking assay, we found also that both MB_CRS6_-13 and MB_CRS6_-15 prevented the formation of CrSAS-6_NL lattices (Supplementary Fig. 4e–f). This was expected in the case of MB_CRS6_-15, given that not even ring assembly occurred (see Fig. 2d). In the case of MB_CRS6_-13, since the co-crystal structure established that monobodies extend above and below the plane defined by pairs of CrSAS-6_N domains when present in the ring polymer (see Supplementary Fig. 3e), it is likely that MB_CRS6_-13 generated a steric clash between superimposed rings, thus preventing stacking.

Taken together, the PORT-HS-AFM and cryo-EM assays lead us to conclude that the three monobodies each have a distinct impact on CrSAS-6 assembly: MB_CRS6_-1 increases slightly ring diameter, MB_CRS6_-13 prevents ring stacking, whereas MB_CRS6_-15 induces higher order oligomers that form linear assemblies instead of ring polymers.

### MB_[CrSAS-6]_-15 induces helical higher order oligomers instead of ring polymers

**Fig. 3:**
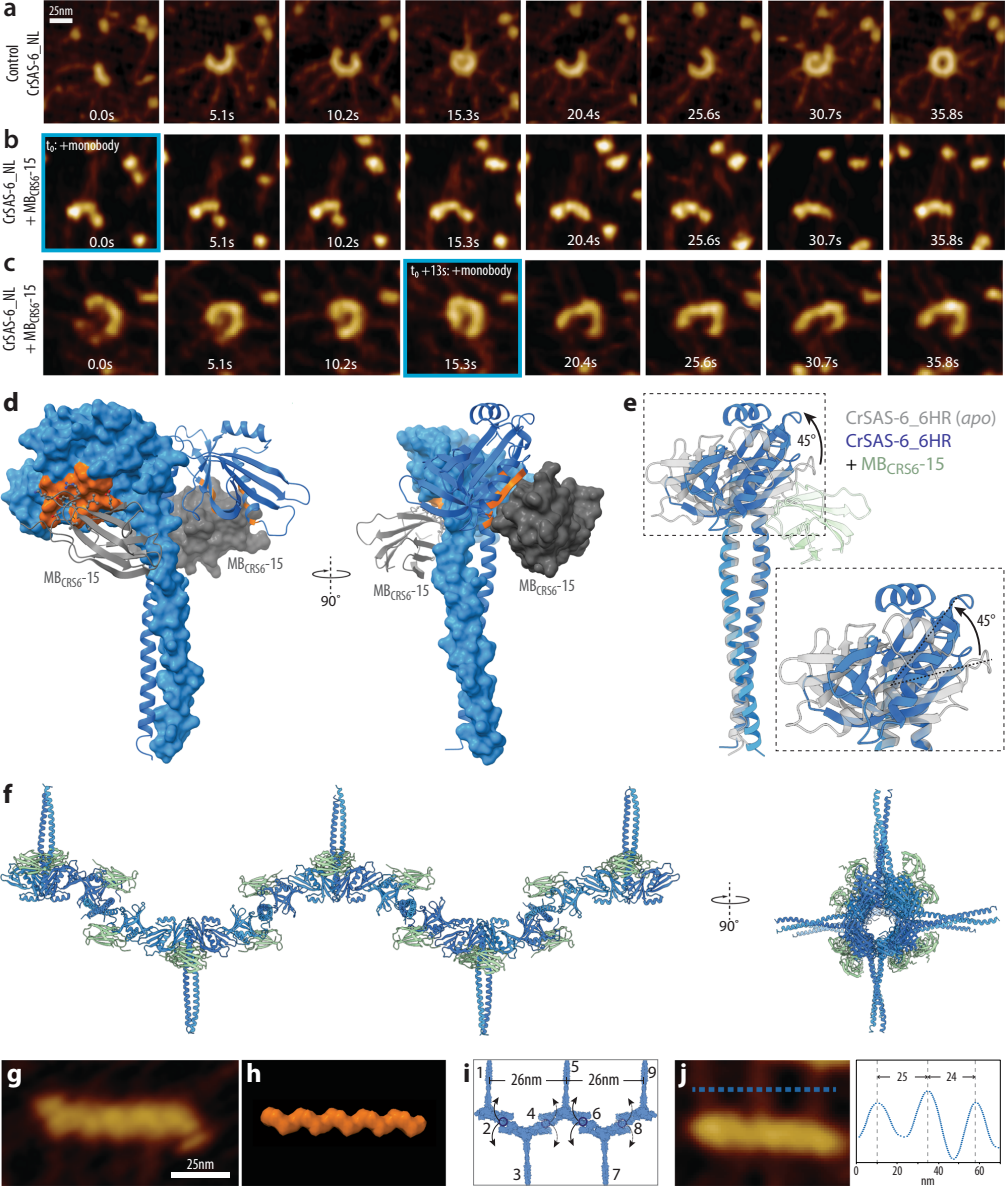
Mechanism of MB_CRS6_-15 inhibition of CrSAS-6 ring formation. **a**-**c** PORT-HS-AFM of CrSAS-6_NL assembly reactions in the absence (**a**) or the presence of MB_CrS6_-15 added at the onset of the assembly reaction (**b**), or during its progression (**c**) (time of addition indicated by blue contour in both cases). Frames with matching effective surface concentration are displayed to monitor the effect of MB_CRS6_-15 on the assembly process. Z scale color bar as in Fig. 2a–d, –0.5 to 10 nm. All measurements were performed at least twice. **d** Structure of CrSAS-6_6HR in surface and ribbon representation (blue), highlighting in orange the residues interacting with MB_CRS6_-15 (gray surface and ribbon representation), viewed from two angles, as indicated. **e** Visualization of conformational change in CrSAS-6_6HR induced by MB_CRS6_-15 (light green); gray shows initial position of the N-terminal domain. Upon MB_CrS6_-15 addition, the N-terminal domains are rotated by ~45° relative to this initial position, which is also highlighted in the higher magnification in the dashed box. **f** The CrSAS-6-6HR higher oligomer formed upon MB_CRS6_-15 addition is expected to be helical, with a ~26 nm pitch and each coiled-coil being rotated by ~90° relative to the neighboring one. Thus, the spiral has an apparent fourfold symmetry. Assemblies observed by PORT-HS-AFM of CrSAS-6_NL upon addition of MB_CRS6_-15 (**g**) resemble a model of the helix predicted by the MB_CRS6_-15 – CrSAS-6_6HR co-crystal structure (**h**). **i, j** Schematic of maximal predicted spoke-to-spoke distance in fourfold helical assembly imposed by MB_CRS6_-15 binding to CrSAS-6_NL (**i**). Arrows indicate potential spoke orientation upon landing on the mica. Example of such spoke-to-spoke distances in PORT-HS-AFM data set determined along the blue dashed line (**j**); Y axis displays relative height.

We investigated further the mechanisms through which MB_CRS6_-15 results in linear assemblies of CrSAS-6_NL higher order oligomers. We conducted PORT-HS-AFM experiments not simply to probe assemblies at equilibrium as reported in Fig. 2a–d, but rather to monitor the kinetics of the assembly reaction. In control conditions, this enabled us to follow the stepwise self-assembly of higher oligomeric species of CrSAS-6_NL up to the formation of ring polymers (Fig. 3a; Supplementary Movie 1, left)^26^. Adding MB_CRS6_-15 at the beginning of the reaction resulted in oligomeric species that lacked the characteristic curved features of native CrSAS-6_NL (Fig. 3b; Supplementary Movie 1, right). Therefore, MB_CRS6_-15 prevents the onset of proper higher order oligomerization. To address whether MB_CRS6_-15 also impairs progression of the assembly reaction once it has been initiated, we started the experiment with CrSAS-6_NL alone and perfused MB_CRS6_-15 in the chamber at a later time point. As shown in Fig. 3c, we found that such addition interrupted the normal course of events and straightened the curvature of higher oligomeric species, which no longer closed into ring polymers. Overall, we conclude that MB_CRS6_-15 prevents both onset and progression of the CrSAS-6_NL self-assembly reaction into rings.

To understand how MB_CRS6_-15 can lead to the formation of linear assemblies of CrSAS-6_NL higher order oligomers, we determined its co-crystal structure with CrSAS-6_6HR to uncover the impact of the monobody on the coiled-coil moiety (Fig. 3d). Crystals of the MB_CRS6_-15 – CrSAS-6_6HR complex diffracted to 3.73 Å and the structure was solved by molecular replacement. The final model was refined to R/R_free_ 22.5/26.7 and the ASU consisted of six CrSAS-6_6HR molecules each bound to one MB_CRS6_-15 (Fig. 3d, Supplementary Fig. 5a, Table 1). The binding mode was identical to that in the co-crystal with CrSAS-6_N, with an overall RMSD of 0.855 Å. Strikingly, the arrangement of molecules in the ASU revealed that MB_CRS6_-15 imparted a major conformational alteration that resulted in each of the head domains within a homodimer to be tilted by ~45° relative to the axis of the coiled-coil (Fig. 3e, Supplementary Fig. 5a–c; Supplementary Movie 2). Overall, this resulted in a ~90° tilt between adjacent homodimers (Supplementary Fig. 5c), which is expected to propagate across higher order oligomers and lead to the formation of a helix with a 26 nm pitch and 4 molecules per turn (Fig. 3f, Supplementary Fig. 5d; Supplementary Movie 3). Accordingly, the linear assemblies generated by CrSAS-6_NL upon addition of MB_CRS6_-15 and monitored by PORT-HS-AFM were helical in nature (Fig. 3g, h). Moreover, we reasoned that upon landing on the mica surface, a CrSAS-6_NL helix might display predictable spacing between spokes emanating from the N-terminal head domains depending on which side of the helix they land, with a maximal distance between spokes of 26 nm given the fourfold screw axis (Fig. 3i). As shown in Fig. 3j, we indeed found examples of CrSAS-6_NL higher oligomeric assemblies with such spacing between spokes emanating from the head domains.

Taken together, these observations demonstrate that MB_CRS6_-15 induces a major conformational shift in CrSAS-6 that leads to the formation of a helix instead of a ring upon higher order oligomerization.

### Monobodies against CrSAS-6 modulate centriole assembly in a cellular context

We set out to test the consequences of expressing the three monobodies in the cellular context. Because repeated attempts at expressing them in *Chlamydomonas* were not met with success, echoing well-known difficulties in stably transforming cells in this organism^43,44^, an alternative strategy was designed. We reasoned that since the monobodies are directed against the CrSAS-6 N-terminal head domain, or that domain plus six heptad repeats of the coiled-coil, a chimeric protein containing the corresponding residues of CrSAS-6 followed by the remaining amino acids of HsSAS-6 might enable us to investigate the impact of the monobodies in human cells (Supplementary Fig. 6a).

We first generated a mini-HsSAS-6 construct using the HsSAS-6 promoter to drive expression of the HsSAS-6 cDNA fused to GFP (Supplementary Fig. 6b). The resulting construct was delivered with lentiviruses and stably integrated into the genome of non-transformed human RPE-1 cells lacking endogenous HsSAS-6, as well as p53^45^, to avoid a possible p53-dependent block in cell cycle progression in response to faulty centrioles^46–49^. We isolated a lentivirus-treated clone in which HsSAS-6-GFP expression levels were similar to those of endogenous HsSAS-6 (Supplementary Fig. 6c), and the fusion protein was likewise regulated across the cell cycle, being maximal toward the end of G2 (Supplementary Fig. 6d)^22^. We similarly generated a cell line stably expressing the chimeric version of the protein, ChSAS-6-GFP. In this case, we selected a cell line with higher levels of the fusion protein to maximize the likelihood that it would be functional and thus enable us to test the impact of the monobodies (Supplementary Fig. 6c). In the background of these cells stably expressing ChSAS-6-GFP, we introduced doxycycline-inducible constructs to express the monobodies (Supplementary Fig. 6e), ascertaining through co-immunoprecipitation that they interacted with ChSAS-6-GFP in the cellular context (Supplementary Fig. 6f).

**Fig. 4:**
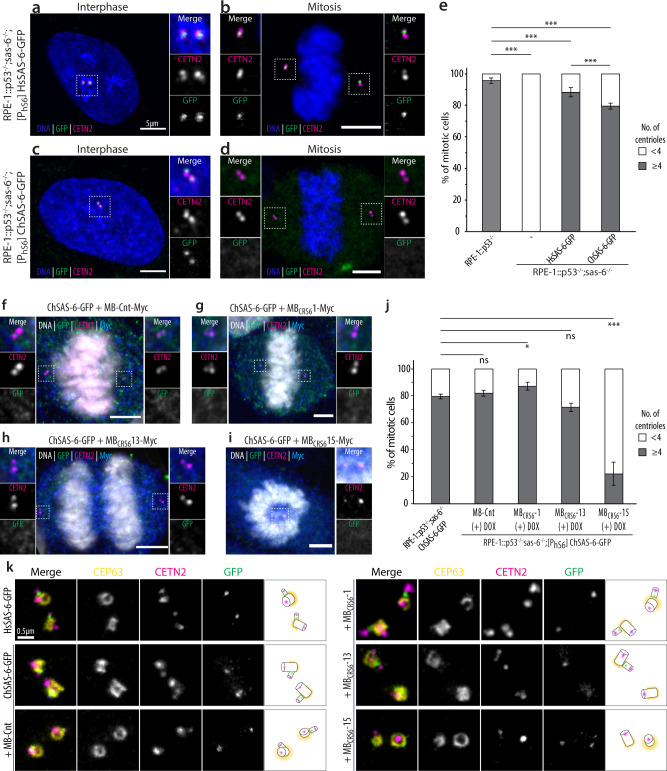
MB_CRS6_-15 impairs centriole assembly in the cellular context. **a**-**d** Confocal images of RPE-1::p53^−/−^;sas-6^−/−^ cells expressing HsSAS-6-GFP (**a**, **b**) or ChSAS-6-GFP (**c**, **d**) in interphase (**a**, **c**) and mitosis (**b**, **d**). Cells were stained with antibodies against Centrin-2 (CETN2; to mark centrioles, magenta) and GFP (to detect HsSAS-6, green), as well as counterstained with a DNA dye (blue). Scale bars: 5 µm. **e** Quantification of mitotic cells of indicated types with <4 or ≥4 Centrin-2 foci. Note that all RPE-1::p53^−/−^;sas-6^−/−^ were entirely lacking centrioles. Number of cells analyzed in at least 3 independent experiments: RPE-1::p53^−/−^ *N* = 406, RPE-1::∆SAS-6 *N* = 275, HsSAS-6-GFP *N* = 527, ChSAS-6-GFP *N* = 634. Statistical analysis was performed using one-tailed chi‐square test (RPE-1::p53^−/−^ versus RPE-1::∆SAS-6 *p* < 0.001; RPE-1::p53^−/−^ versus HsSAS-6-GFP *p* = 0.000052; RPE-1::p53^−/−^ versus ChSAS-6-GFP *p* < 0.001; HsSAS-6-GFP versus ChSAS-6-GFP *p* < 0.001). *** *p* < 0.001, ** *p* < 0.01, * *p* < 0.05, ns *p* ≥ 0.05. Un-binned data are shown in Supplementary Fig. 7a. **f–i** Confocal images of mitotic RPE-1::p53^−/−^;sas-6^−/−^ cells expressing HsSAS-6-GFP or ChSAS-6-GFP and induced to express the indicated monobodies. Cells were stained with antibodies against Centrin-2 (CETN2; to mark centrioles, magenta) and GFP (to detect SAS-6, green), as well as counterstained with a DNA dye (blue). Scale bars: 5 µm. **j** Quantification of mitotic cells of indicated types with <4 or ≥4 Centrin-2 foci. Number of cells analyzed in at least 3 independent experiments: ChSAS-6-GFP *N* = 634, ChSAS-6-GFP + MB-Cnt *N* = 326, ChSAS-6-GFP + MB_CRS6_-1 *N* = 270, ChSAS-6-GFP + MB_CRS6_-13 *N* = 325, ChSAS-6-GFP + MB_CRS6_-15 *N* = 273. Statistical analysis was performed using one-tailed chi‐square test (ChSAS-6-GFP versus ChSAS-6-GFP + MB-Cnt *p* = 0.198, ChSAS-6-GFP + MB_CRS6_-1 *p* = 0.007; ChSAS-6-GFP versus ChSAS-6-GFP + MB_CRS6_-13 *p* = 0.07; ChSAS-6-GFP versus ChSAS-6-GFP + MB_CRS6_-15 *p* < 0.001). *** *p* < 0.001, ** *p* < 0.01, * *p* < 0.05, ns *p* ≥ 0.05. Un-binned data are shown in Supplementary Fig. 7b. **k** STED images of RPE-1::p53^−/−^;sas-6^−/−^ cells treated with a double thymidine block (i.e., arrested at the G_1_/S transition), and expressing the constructs indicated on the left. Cells were stained with antibodies against CEP63 (to mark the torus encircling the proximal part of the resident centriole, yellow), Centrin-2 (to mark centrioles, magenta), and GFP (to detect SAS-6, green). Boxes on the right are schematics of centriole orientation, along with corresponding distributions. All measurements were performed twice, with at least 10 cells imaged in each experiment.

We set out to test the ability of ChSAS-6-GFP to sustain centriole formation in human cells, using immunofluorescence analysis and confocal microscopy. Cells were examined during interphase and mitosis for the presence of the centriolar marker Centrin-2 and of the fusion protein, using antibodies that recognize an epitope in the C-terminal half of HsSAS-6 present in the chimeric construct (Fig. 4a–e, Supplementary Fig. 7a). We found that ~95% of control mitotic cells exhibited ≥4 foci bearing the centriolar marker Centrin-2 (Fig. 4e, Supplementary Fig. 7a). Moreover, as expected, Centrin-2 foci were never detected in the parental cells lacking HsSAS-6 and p53 (Fig. 4e, Supplementary Fig. 7a). We found that expression of wild-type HsSAS-6-GFP in this background led to ≥4 Centrin foci in ~85% of mitotic cells (Fig. 4b, e, Supplementary Fig. 7a). Importantly, we established that this was the case also in ~75% of mitotic cells expressing ChSAS-6-GFP (Fig. 4d, e, Supplementary Fig. 7a). Moreover, we found that whereas GFP foci were present both in interphase and mitotic cells expressing HsSAS-6-GFP, GFP foci were usually present only during interphase in cells expressing ChSAS-6-GFP, indicative of impaired protein maintenance (Fig. 4a–d, Supplementary Fig. 7c, d). We found also that cells lacking endogenous HsSAS-6 and expressing ChSAS-6-GFP sustained robust bipolar spindle formation, much like upon expression of HsSAS-6-GFP (Supplementary Fig. 7b). Overall, we conclude that a chimeric protein comprising an N-terminal fragment of the *Chlamydomonas* CrSAS-6 protein followed by sequences of the human protein HsSAS-6 is recruited to the emerging procentriole, but is not properly maintained, yet can sustain centriole assembly at least in part.

This experimental setting enabled us to test the impact of doxycycline-mediated expression of MB_CRS6_-1, MB_CRS6_-13 and MB_CRS6_-15 (Fig. 4f-j, Supplementary Fig. 7a, e–h). We found that expression of a non-binding control monobody^50^ had a marginal effect, with ~80% of mitotic cells harboring ≥4 Centrin foci (Fig. 4f, j). Although MB_CRS6_-1 had little impact on the overall fraction of mitotic cells harboring ≥4 Centrin foci (Figs. 4g, 4j), it increased the fraction of those with more than 4 Centrin foci (Supplementary Fig. 7a), perhaps reflecting more stable ring assemblies. Expressing MB_CRS6_-13 lead to a slight decrease to ~70% in the fraction of mitotic cells with ≥4 Centrin foci (Fig. 4h, j). We noted that this monobody expressed at lower levels than the other two (see Supplementary Fig. 6e, f), which might explain the weak impact observed in cells. Strikingly, our analysis also uncovered a pronounced failure in centriole formation upon expressing MB_CRS6_-15, with only ~25% of mitotic cells harboring ≥4 Centrin foci (Fig. 4i, j). As expected, this was often accompanied by monopolar spindle assembly (Fig. 4i, Supplementary Fig. 7b, h). We sought to corroborate these findings with STED super-resolution microscopy, analyzing cells subjected to a double thymidine block to analyze procentriole assembly onset (Fig. 4k). We found that HsSAS-6-GFP, ChSAS-6-GFP, as well as ChSAS-6-GFP in the presence of control monobodies, of MB_CRS6_-1 or of MB_CRS6_-13, were typically present next to the proximal part of the resident centriole marked by the torus protein Cep63, with 4 Centrin foci in their vicinity (Fig. 4k). When cells expressed MB_CRS6_-15, some ChSAS-6-GFP was present as well, indicative of proper recruitment to the torus (Fig. 4k). Strikingly, however, only 2 Centrin foci were typically detected in the vicinity, demonstrating a failure in centriole assembly (Fig. 4k).

Taken together, we conclude that MB_CRS6_-15 turn SAS-6 higher oligomeric species into a helix, severely impairing centriole formation in the cellular context.

## Discussion

The centriole is essential for forming cilia and centrosomes. Thereby, this organelle is fundamental for proper cell physiology and human health, as is clear also from the fact that mutations in genes governing centriole structure, number or function lead to a range of human diseases^3–5,7,51^. Despite important progress in recent years, understanding of the mechanisms governing this dynamic process remains incomplete, in part because of the paucity of reagents to dissect and modulate distinct steps of organelle biogenesis. Here, we identified and characterized the mechanism of action of three monobodies that each tune in a specific manner the assembly of SAS-6, a protein critical for the onset of centriole biogenesis, revealing the power of this class of reagents in deciphering an evolutionarily conserved self-organizing process.

Proteins and protein-protein assemblies can be targeted with several classes of reagents^52–55^. Small molecule compounds can target minute pockets and interfaces with high selectivity. For instance, in the context of centriole assembly, the small molecule Centrinone targets selectively and with sub-nanomolar K_D_ the ATP binding pocket of the Plk4 kinase, which plays a critical role in defining the site from which the procentriole emerges^11^. However, small molecule compounds are less well suited to target larger binding interfaces and protein-protein interactions in oligomeric assemblies. In the case of centriole assembly, this is exemplified by the fact that the small molecules that have been identified against SAS-6 from *C. elegans*^56^ and from *Leishmania major*^57^ require mM concentrations to prevent oligomerization. Several alternative approaches can be pursued to overcome this limitation, including de novo peptide design and selection of protein binders to the target of interest. Such approaches sample a large interaction space and include the raising of antibodies, as well as the selection of smaller protein-based agents such as affimers^53^, nanobodies^58^ or the monobodies deployed here. Our work demonstrates that monobodies can be developed as reagents to target a protein undergoing self-assembly into higher order oligomers. This holds important potential not only as tool compounds for research, but also as starting point for novel therapeutic approaches.

**Fig. 5:**
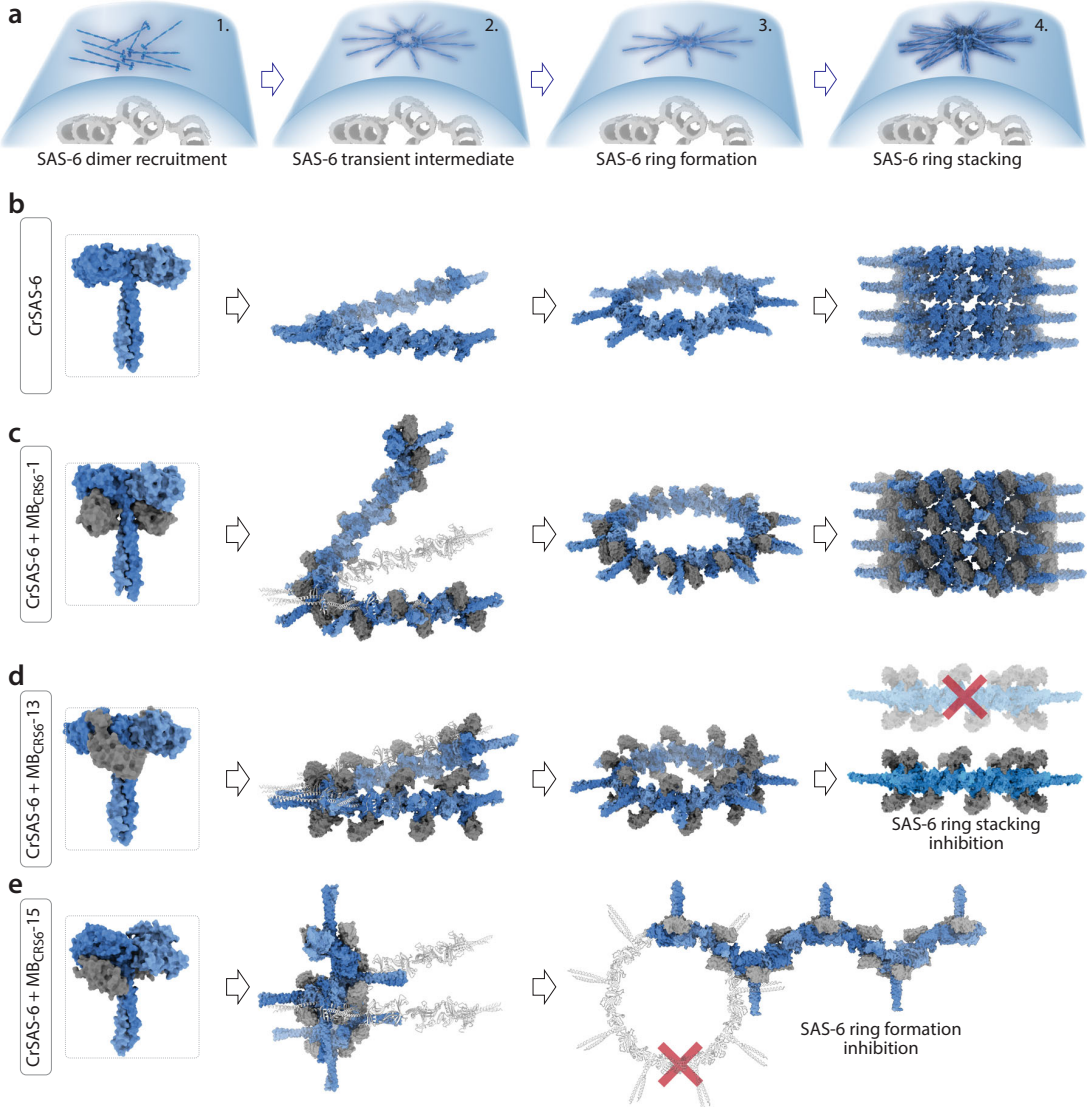
Monobodies against CrSAS-6 impair distinct steps of the assembly reaction. **a, b** Schematic of procentriole formation in normal conditions. SAS-6 is recruited to a torus surrounding the resident centriole (1, curved blue surface, part of resident centriole viewed in cross section), where procentriole assembly begins by SAS-6 homodimers polymerizing into higher order oligomers, including a postulated intermediate helical state (2), which then closes into a ring polymer (3). Thereafter, such ring polymers stack, forming the cartwheel element at the root of centriole assembly (4). **c**–**e** Model of the consequences of monobody binding for the SAS-6 assembly reaction. MB_CRS6_-1 induces an intermediate state with a higher pitch, which leads to ring polymers with a slightly higher mean diameter (**c**); MB_CRS6_-13 is positioned above and below the plane of the SAS-6 ring polymer, which interferes with stacking (**d**); MB_CRS6_-15 causes a drastic conformational shift leading SAS-6 to polymerize into a helix with a fourfold screw axis, which prevents ring polymer formation (e).

Structural, biophysical, and cellular analyses together revealed that the three monobodies characterized here each modulate in a distinct manner the function of the centriole cartwheel protein SAS-6 (Fig. 5). Normally, homodimers of SAS-6 are recruited to a single location on the torus surrounding the resident centriole. There, SAS-6 homodimers undergo higher order oligomerization and self-assemble into ring polymers before stacking on top of another with ~4.5 nm mean periodicity (Fig. 5a, b)^31^. MB_CRS6_-1 binding to SAS-6 does not impact its ability to form rings or stacks (Fig. 5c). However, MB_CRS6_-1 alters the pitch of a putative transient helical SAS-6 intermediate. We propose that the resulting ring polymer adopts a slightly increased diameter on average to compensate for the strain imparted by the increased pitch; this has no detectable detrimental consequence in the cellular context. Moreover, MB_CRS6_-1 might stabilize the SAS-6 ring polymer, since the head-to-head interface is present in the co-crystal structure despite the F145E mutation, in line with the higher number of centrioles observed in cells upon MB_CRS6_-1 expression.

MB_CRS6_-13 does not interfere with SAS-6 ring polymer formation either. However, being positioned slightly above and below the plane of the ring polymer, MB_CRS6_-13 prevents stacking in vitro, thus targeting a distinct step of the assembly reaction (Fig. 5d). Intriguingly in the light of this result, MB_CRS6_-13 expression is of little consequence *in cellulo*. Although this may be due to the low expression levels, an intriguing alternative is that a single SAS-6 ring polymer suffices to jump start centriole assembly in the cellular context. Moreover, cellular factors besides HsSAS-6 might contribute to stacking and enable this process to occur despite the intercalating MB_CRS6_-13.

Strikingly, the third monobody analyzed in detail here, MB_CRS6_-15, has a profound impact on SAS-6, leading to the formation of helical higher order oligomers instead of ring polymers, and to severely impaired centriole assembly in human cells (Fig. 5e). Why is such a helical configuration imposed by MB_CRS6_-15? Computational modeling based on the CrSAS-6 crystal structures raises the possibility that a helix with a shallow pitch might be present transiently before ring closure^25^. Moreover, SAS-6 has been proposed to form a spiral in the nematode *C. elegans*, in this case with 9 homodimers per two turns^59^. By contrast, MB_CRS6_-15 imposes a 4-fold screw axis to CrSAS-6 that is not compatible with the signature ninefold symmetry of centrioles. Interestingly, sixfold and twofold screw axis helices are present in oligomers of the XRCC4/XLF complex^60^ and CCDC61^61^, respectively; both XRCC4 and CCDC61 proteins exhibit structural relatedness with the SAS-6 protein family, yet do not participate in centriole assembly. Together, the above considerations lead us to propose that MB_CRS6_-15 binding stabilizes a transient state of CrSAS-6 and acts as a molecular lever to induce a conformational change into a fourfold screw helix. As a result, MB_CRS6_-15 prevents SAS-6 ring polymer formation, cartwheel assembly and centriole biogenesis, demonstrating that proper architecture of SAS-6 oligomers is critical for function.

In conclusion, our work illustrates how monobodies serve as powerful reagents to dissect and deepen understanding of specific steps in a complex assembly reaction such as that leading to formation of the cartwheel at the root of centriole biogenesis.

## Methods

### Cloning, protein expression and purification

The CrSAS-6_6HR and CrSAS-6_N constructs for monobody selection were prepared by introducing a DNA fragment encoding respectively CrSAS-6 residues 1-159 and 1-226 in the pHFT vector using BamHI and XhoI sites. The resulting constructs produce proteins with an N-terminally located 6xHis tag followed by an AVI-tag and a TEV protease cleavage site. Monobody constructs for protein expression were also cloned in pHFT, using BamHI and XhoI sites. For production of CrSAS-6_NL, a DNA fragment encoding residues 1-503 was cloned using Gibson assembly in a pFLOAT2-His^62^ vector providing an N-terminally located 6xHis tag followed by a PreScission Protease cleavage site. All oligonucleotide primers are listed in Supplementary Table 2.

Recombinant protein expression was performed in the *Escherichia coli* strain BL21(DE3) in LB medium. Bacteria were grown at 37 °C to an OD_600nm_ of 0.7 in LB medium containing kanamycin. Following a cold shock on ice, protein expression was induced at 18 °C by the addition of 0.3 mM IPTG and allowed to proceed for ~16 h. For production of biotinylated proteins used for monobody selection, CrSAS-6_6HR or CrSAS-6_N were co-expressed in *E. coli* BL21 (DE3) cells with BirA^39^. Upon induction, 1 mg/mL biotin (Merck) was added to the culture to achieve biotinylation in vivo. Bacterial cells were collected by centrifugation at 5000 × *g* for 10 min, and the pellet washed with ice cold PBS, flash frozen and stored at −20 °C until processing.

Bacterial cell pellets were resuspended in lysis buffer containing 50 mM Tris-HCl pH 8.0, 400 mM NaCl, 20 mM Imidazole pH 8.0, 3 mM β-mecraptoethanol (β-ΜΕ), 50 Units DNAseA, 3 mM MgCl_2_, protease inhibitors (Complete EDTA-free, Roche), 1% v/v Tween 20, and lysed by lysozyme treatment and sonication. Lysates clarified from cellular debris were loaded on a 5 mL HisTrap column (GE Healthcare) at 4 °C according to the manufacturer’s instructions. The best fractions of eluted proteins were dialyzed overnight against 50 mM Tris-HCl pH 8.0, 400 mM NaCl, 3 mM β-mecraptoethanol (β-ΜΕ). For production of proteins without tag used for crystallization and affinity measurement experiments, protease (TEV or PreScission protease) was added at this stage. An additional step to remove un-cleaved protein was introduced following tag cleavage by protease. Finally, proteins were concentrated and further purified with SEC equilibrated in 25 mM Tris pH 8.0, 150 mM NaCl and 3 mM DTT. All purified proteins were concentrated and snap-frozen in liquid nitrogen and stored at −20 °C.

### Monobody selection process

Selection was performed using biotinylated purified CrSAS-6_6HR and CrSAS-6_N. The monobody selection process has been described in detail previously^39^. Briefly (Supplementary Fig. 1b), several rounds of phage display were performed to select a pool of binders with moderate affinities (~1–4 weeks). Thereafter, the DNA of the selected monobodies was isolated, and loops were PCR amplified with extension primers in preparation for yeast display selection. Yeast cells were transfected with amplified loop fragments and with a yeast vector backbone for homologous recombination (~1 week). Several rounds of yeast display were then performed by sequentially lowering the concentration of the target until only a few clones were left in the pool, and lower concentration of target no longer led to clones with better affinity (~2–4 weeks). Single clones were plated on CAA-agar plates and monobody DNA fragments isolated by colony PCR before sequencing. Yeast display was performed using antibodies to detect the V5 tag of the monobodies displayed on the yeast surface, and Streptavidin-Dylight650 or Neutravidin-Dylight650 to detect binding to the biotinylated target.

Nine of the 14 monobodies thus selected could be purified in sufficiently high yield to be tested using the cryo-EM stacking assay (see below). Seven of these exhibited decoration on the outside of the SAS-6 stack of rings, as exemplified by MB_CRS6_-1, whereas MB_CRS6_-13 and MB_CRS6_-15 did not exhibit such decoration. Two of the 7 monobodies that exhibit decoration on the outside were processed for crystallization trials, but only MB_CRS6_-1 gave diffracting crystals.

### Monobody specificity and affinity measurements

The yeast binding assay has been performed as described^39^. Biotinylated targets of different concentrations were incubated with single yeast clones at an OD_600_ of 1, with a final volume of 20 µL in a 96 well format for 60 min, stained with FITC conjugated antibodies to detect yeast display levels and with Dylight650 conjugated Streptavidin to detect target binding. Single cells displaying monobodies were gated and target binding determined using a 640 nm laser line. Binding curves were determined by plotting mean fluorescent values against target concentration.

The affinity of the purified monobodies toward target proteins was determined by isothermal titration calorimetry at 25 °C with a MicroCal ITC200. All samples were extensively dialysed against sample buffer (30 mM Tris pH 7.5, 150 mM NaCl pH 7.5) before measurement. The affinity (K_D_), binding enthalpy (∆H) and stoichiometry (n) were determined using the MicroCal software. In general, concentration of protein in the sample syringe was chosen to be ten times higher than that of the protein in the sample cell as we expected 1:1 complex formation of monobody to CrSAS-6. For all ITC experiments, monobodies in the syringe were titrated against CrSAS-6 in the cell, except for MB_CRS6_-4 where high enough concentration (>100 µM) could not be reached. A full description of sample concentrations and binding constants for ITC experiments is provided in Supplementary Table 1.

### Crystallisation and structure determination

Complexes of monobodies with CrSAS-6_N or CrSAS-6_6HR were prepared prior to crystallisation screening by combining CrSAS-6 with a 2x molar excess of monobodies followed by SEC to purify the complex. Crystals were obtained using the sitting drop vapor diffusion technique at 18 °C. A Mosquito robot (TTP LabTech) was used to set up 200 nl size drops with 1:1 and 1.3:0.7 ratios of protein to mother liquor. Initial hits were optimised, resulting in the following crystallisation conditions: 15% (w/v) PEG 4000, 0.8 M sodium formate, 0.1 M Tris pH 8.5 for MB_CRS6_-1 – CrSAS-6_6HR-[F145E]; 15% (w/v) PEG 4000, 0.2 M magnesium chloride hexahydrate, 0.1 M Tris pH 8.5 for MB_CRS6_-13 – CrSAS-6_N; 0.3 M Sodium acetate trihydrate, 10% (w/v) PEG8000, 10% (w/v) PEG 1000, 4% (v/v) 1,1,1,3,3,3-Hexafluoro-2-propanol and 0.1 M Tris pH 8.5 for MB_CRS6_-15 – CrSAS-6_N; and 40% (v/v) MPD, 5% (w/v) PEG 8000, 0.1 M MES pH 6.5, 0.4 M DL-Glutamic acid monohydrate for MB_CRS6_-15 – CrSAS-6_6HR. Following cryo-protection with glycerol for the co-crystals with MB_CRS6_-1 and MB_CRS6_-13, crystals were vitrified in liquid nitrogen and data were collected at the Swiss Light Source (PSI, Villingen, Switzerland) (Table 1).

Structures were solved with molecular replacement using Phenix-Phaser^63^ for all structures except MB_CRS6_-15 – CrSAS-6_N where Molrep^64^ was used. Structures were refined using PHENIX.refine^63^ with TLS parameters and NCS restraints. Crystallographic data processing and refinement statistics are provided in Table 1. Model quality was assessed by MolProbity^65^ and associated data have been deposited in the RCSB databank under accession numbers 6ZZC, 6ZZD, 6ZZG and 6ZZ8. For graphical representation, we used ChimeraX^66^.

Three copies of each molecule are present in the ASU of MB_CRS6_-1 – CrSAS-6_6HR-[F145E] crystal structure. Several residues at both N- and C-termini were disordered and thus not observed in the electron density map. Two copies of each molecule are present in the ASU of MB_CRS6_-13 – CrSAS-6_N, while the biological unit is assembled through the symmetry related molecules that interact with CrSAS-6-N. The His^6x^-AVI-TEV tag of MB_CRS6_-13 was not cleaved and thus some additional amino acids have been modeled in the electron density. In the ASU of MB_CRS6_-15 – CrSAS-6_N crystal structure, there are six molecules of CrSAS-6-N in a head-to-head dimeric arrangement, with each molecule of MB_CRS6_-15 interacting with one molecule of CrSAS-6_N. There are six copies of MB_CRS6_-15 – CrSAS-6_6HR 1:1 complex in the co-crystal structure. The last ~30 residues (31 aa, 28 aa, 30 aa, 28 aa, 24aa, 23 aa for chain A, B, C, D, E, F, respectively) of the coiled-coil are not visible in the electron density.

### Photothermally actuated off resonance tapping high-speed atomic force microscopy (PORT-HS-AFM)

PORT-HS-AFM of CrSAS-6_NL was performed as described^26^. In short, for equilibrium measurements, a 10 µL droplet of sample diluted to 11-16 nM was deposited on freshly cleaved mica, covered to avoid evaporation and incubated for ~30 min at 4–10 °C. Due to the heat generated by the readout electronics of the PORT-HS-AFM system, incubation and imaging are performed in a low vibration cooler^26^. Imaging was performed following a thorough rinsing with imaging buffer (150 mM KCl, 20 mM Tris-HCl pH 8.0) at 100 Hz, covering 1024 pixels × 512 lines, corresponding to 2.56 s frame^–1^. For imaging of CrSAS-6_NL complexed with monobodies, the concentrated sample was diluted in imaging buffer and monobodies to achieve a minimum of 70% binding, factoring in the K_D_ measured by ITC. To that end, MB_CRS6_-1, MB_CRS6_-13 and MB_CRS6_-15 were added to a final concentration of 6.8 µM, 14.5 µM, and 12.9 µM, respectively. For PORT-HS-AFM imaging of the entire assembly reaction, 5 µL of CrSAS-6_NL or preassembled CrSAS-6_NL-MB_CRS6_-15 complex was injected with a Hamilton syringe in the liquid cell already containing 70 µL of buffer, reaching a final CrSAS-6_NL concentration of 44 nM. For the experiment where MB_CRS6_-15 was added at a later time point, an additional 2 µL of concentrated monobodies were injected after ring polymers had been observed on the surface. PORT-HS-AFM movies were processed with Gwyddion software followed by denoise filter (1 pixels). All equilibrium PORT-HS-AFM measurements were performed at least twice, with at least 5 field of view imaged in each experiment. Frames from PORT-HS-AFM measurements of CrSAS-6_NL reported in^67^ were used as control. Time-lapse PORT-HS-AFM measurements were performed twice at a single position followed by imaging a large field of view (1200 × 1200 nm) at a secondary location to verify that the structures observed were not caused by the imaging.

For the analysis of the height, ring particles were extracted from the PORT-HS-AFM data set. Each ring was fitted with a circle to determine the radius and center, from which 40 radial profiles were extracted across the ring circumference, determining for each radial profile the highest (maximum) and lowest height (baseline). The height for each position on the ring is then calculated as height = maximum - baseline. Thereafter, for each ring, the difference between the higher and the lower height value is determined and reported as “height difference”.

### In vitro cryo-electron microscopy (EM) stacking assay

The cryo-EM stacking assay was performed as described previously with small modifications^42^. Briefly, 10 µL of CrSAS-6_NL at 40 µM or CrSAS-6_NL with a 50% molar excess of monobodies were set for dialysis overnight at 4 °C into 10 mM K-PIPES pH 7.2 using a 3 kDa MWCO slide-A-lyzer mini dialysis unit (Pierce). From the recovered sample, 5 µL were further used for preparing EM grids using a Vitrobot (ThermoFisher Scientific). The sample was applied on a Lacey carbon film grid (300 Mesh, EMS), incubated for 60 s at 5 °C, then blotted for 3 s with -15 blot force and vitrified in liquid ethane.

EM was performed on a Tecnai F20 field emission gun electron microscope (ThermoFisher Scientific) operating at 200 kV and equipped with an Eagle camera 4096 × 4096 or a Falcon 2 direct electron detector. All images were recorded at 29,000x magnification with a total dose of 20e/Å^2^ (final pixel size 0.225 nm and 0.349 nm for Eagle and Falcon2, respectively; −2,5 μm defocus). Images on direct electron detector were collected in 10 fractions and automatically aligned by the Epu software (ThermoFisher Scientific).

All images were further analysed with Scipion software^68^. Particles containing top views of CrSAS-6 stacks of rings were picked with RELION2.0^69^. Particles were classified with RELION2.0^69^ and Eman2^70^. For ring diameter measurements, a line scan of aligned particles was performed with FiJi^71^. Plots were then analysed with MATLAB, identifying peaks on plot profiles with the findpeak function; ring diameter was determined as the peak-to-peak distance.

### Generation of expression vectors for in cellulo experiments

Chimeric SAS-6 was created by amplifying regions encoding respectively aa 1-204 of CrSAS-6 and aa 191-657 of HsSAS-6. The DNA fragments were cloned sequentially in the pET30a vector using respectively KpnI and SacI sites, or SacI sites. Chimeric SAS-6 was then subcloned in pENTR1A-eGFP vector using SpeI sites. For simplicity, eGFP is referred as GFP elsewhere in the manuscript. A cDNA encoding HsSAS-6 was cloned into pENTR1A-GFP following the same procedure, using AgeI sites instead of SpeI sites.

For the creation of SAS-6 expression constructs under the control of endogenous promoter, we first created a basic lentiviral plasmid by removing the promoter, the Gateway cloning site and GFP from hPGK-GW-IRES-GFP (Addgene). Then, HsSAS-6-GFP or Chimeric SAS-6-GFP (hereafter ChSAS-6-GFP) were cloned into this vector using XhoI and NheI sites. A genomic region 1034 bp in length upstream of the HsSAS-6 open reading frame (GRCh38, *Homo sapiens* Chromosome 1: 100132815-100133848) was amplified and inserted into the plasmid to act as a native promoter.

Monobodies were cloned into a modified pENTR1A vector to include Myc-tag at the C-terminus using BP Clonase reactions (ThermoFisher Scientific). Monobody H4A_Y87A_ was used as a non-binding control monobody^50^. Entry vectors were then used in LR Clonase reactions (ThermoFisher Scientific) with pCW57.1 (Addgene) to produce expression vectors. All clones were sequence-verified.

### Cell culture

hTERT-RPE-1 cells (ATCC, hereafter RPE-1), RPE-1::p53^−/−^ or RPE-1::p53^−/−^;sas-6^−/−^ (both gifts from Bryan Tsou, MSKCC, New York City, USA) were cultured in DMEM/F-12 (ThermoFisher Scientific) with 10 % FBS (Merck), 0.2 mM sodium pyruvate (ThermoFisher Scientific), MEM non-essential amino acids (ThermoFisher Scientific) and supplemented with 10 μg/ml puromycin (Merck) for selection of cells stably expressing monobodies. HEK293T cells (ThermoFisher Scientific) were cultured in DMEM and used for lentivirus production. In brief, HEK293T cells were co-transfected using Lipofectamine 3000 (ThermoFisher Scientific) with a lentiviral transfer plasmid encoding either HsSAS-6-GFP, ChSAS-6-GFP or monobodies, as well as with psPAX2 and pMD2.G (both Addgene). After overnight incubation, the medium was replaced and cells grown for an additional 48 h. Lentivirus containing medium was recovered and centrifuged for 15 min at 1000 × *g* to remove cell debris, and the supernatant used to transduce RPE-1::p53^−/−^;sas-6^−/−^ cells. Several days following lentivirus transduction, clonal isolation of cells expressing HsSAS-6-GFP or ChSAS-6-GFP was performed to identify cells with low GFP levels and physiological number of centrioles. The ChSAS-6-GFP expressing clone was further transduced with lentiviruses to produce stably integrated cell lines that also express monobodies. Following transduction, cells were incubated with selective medium containing puromycin until control non-transduced cells were dead. For monobody expression, 1 μg/ml doxycycline (Merck) was used to induce transgene expression.

Expression levels were verified with Western blot analysis of cell lysates. Primary antibodies were diluted in 5% milk TBS-T. Primary antibodies were mouse anti-SAS-6 (Santa Cruz Biotechnology Cat# sc-81431), mouse anti-Cyclin B1 (Santa Cruz Biotechnology Cat# sc-166757), both 500-fold diluted, as well as rabbit anti-Myc-tag (Abcam Cat# ab9106), rabbit anti-β-tubulin (Novus Cat# NB600-936SS), rabbit anti-GFP (a gift from Viesturs Simanis, EPFL, Lausanne, Switzerland), all 1000-fold diluted, and mouse anti-α-tubulin (Sigma-Aldrich Cat# T9026), 5000-fold diluted. Secondary antibodies were anti-rabbit Alex Fluor 680 (ThermoFisher Scientific Cat# A27042) and anti-mouse DyLight 800 (ThermoFisher Scientific Cat# SA5-35521) used at 10,000-fold dilution.

### Immunofluorescence analysis

Cells were grown on glass coverslips and fixed in methanol for 7 min at −20 °C. Cells were permeabilized using 0.05 % (v/v) Tween 20, washed in PBS and 0.05 % (v/v) Tween 20, before blocking for 1 h in PBS supplemented with 0.05 % (v/v) Tween 20, and 3% BSA. All antibodies were diluted in the blocking solution and incubated for either 1 h at room temperature or ∼12 h at 4 °C. Primary antibodies were mouse anti-Centrin-2 (Millipore Cat# 04-1624), rabbit anti-hPOC5 (Abgent Cat# AP10821b), rabbit anti-CEP63 (Millipore Cat# 06-1292), rabbit anti-Myc-tag (Abcam Cat# ab9106), mouse anti-PCNA (Santa Cruz Biotechnology Cat# sc-56), chicken anti-GFP (Abcam Cat# ab13970), rabbit anti-GFP (a gift from Viesturs Simanis, EPFL, Lausanne, Switzerland), rabbit anti-α-tubulin coupled with Alexa Fluor 647 (Abcam Cat# ab190573), and anti-GFP nanobody coupled with Alexa Fluor 488. All primary antibodies were diluted 1000-fold, except Centrin 2 and CEP63, which were diluted 2000-fold. Secondary antibodies were goat anti-rabbit Alexa Fluor 488 (ThermoFisher Scientific, Cat# A-11034), goat anti-Chicken Alexa Fluor 488 (ThermoFisher Scientific, Cat# A-11039), goat anti-mouse Alexa Fluor 568 (ThermoFisher Scientific, Cat# A-11004), donkey anti-Mouse Alexa Fluor 594 (Abcam, Cat# ab150112), goat anti-Rabbit Atto647N (HyperMol Cat# 2318-250UG). All secondary antibodies were diluted 1000-fold for samples prepared for confocal microscopy and 500-fold for samples prepared for STED microscopy. Coverslips were washed three times after each antibody incubation, and further incubated with 1 μg/ml Hoechst in PBS prior to mounting in Fluoromount-G (ThermoFisher Scientific) for confocal or in PBS-90% glycerol-4% N-propyl gallate for STED microscopy.

Confocal imaging was carried out using a Zeiss LSM 700 microscope with a Plan-Apochromat 63× oil-immersion objective, NA 1.40. Z sections were imaged at an interval of ∼0.2 μm. STED imaging was carried out using a Leica TCS SP8 STED 3X microscope with a 100x oil objective (Leica HCX PL APO 100x/1.4 Oil). Fluorophores were excited at the optimal wavelength by an 80 MHz pulsed white light laser (470–670 nm), allowing time gating of fluorescence lifetimes. A depletion laser at 775 nm was used for Atto647N and Alexa Fluor 594, and at 592 nm for Alexa Fluor 488. Z sections were imaged at an interval of ∼0.135 μm using hybrid spectral detectors with a final pixel size of 15.2 × 15.2 nm. All images shown are maximum-intensity projections and were processed using FiJi^71^, maintaining relative intensities within a series.

## Supplementary information

Supplementary Information

Peer Review File

Description of Additional Supplementary Files

Supplementary Movie 1

Supplementary Movie 2

Supplementary Movie 3

## Data Availability

Data supporting the findings of this manuscript are available from the corresponding authors upon reasonable request. A reporting summary for this Article is available as a Supplementary Information file. Source data are provided with this paper. The structures and corresponding structure factors have been deposited into the Protein Data Bank with the PDB accession codes: PDB 6ZZC [10.2210/pdb6ZZC/pdb] PDB 6ZZD [10.2210/pdb6ZZD/pdb] PDB 6ZZG [10.2210/pdb6ZZG/pdb] PDB 6ZZ8 [10.2210/pdb6ZZ8/pdb]. Source data are provided with this paper.

## Source data

Source Data
