# Supplementary material for: Tuning SAS-6 architecture with monobodies impairs distinct steps of centriole assembly
Source: Nat Commun. 2021 Jun 21;12:3805. doi: 10.1038/s41467-021-23897-0 (PMC8217511; doi:10.1038/s41467-021-23897-0)
Supplement: Supplementary file 1 — Supplementary Information [file 41467_2021_23897_MOESM1_ESM.pdf]

Supplementary information

Tuning SAS-6 architecture with monobodies impairs distinct steps of centriole assembly

Georgios N. Hatzopoulos, Tim Kükenshöner, Niccolò Banterle, Tatiana Favez, Isabelle Flückiger, Virginie Hamel, Santiago Andany, Georg E. Fantner, Oliver Hantschel & Pierre Gönczy

Correspondence: P.G. (email: pierre.gonczy@epfl.ch) or to O.H. (email: oliver.hantschel@uni-marburg.de).

<u>Table of contents:</u>

Supplementary figure 1 - pages 2-3
Supplementary figure 2 - page 4
Supplementary figure 3 - page 5
Supplementary figure 4 - page 6-7
Supplementary figure 5 - page 8
Supplementary figure 6 - page 9
Supplementary figure 7 - page 10-11
Supplementary table 1 - page 12
Supplementary table 2 - page 13
Supplementary source data - page 14-15

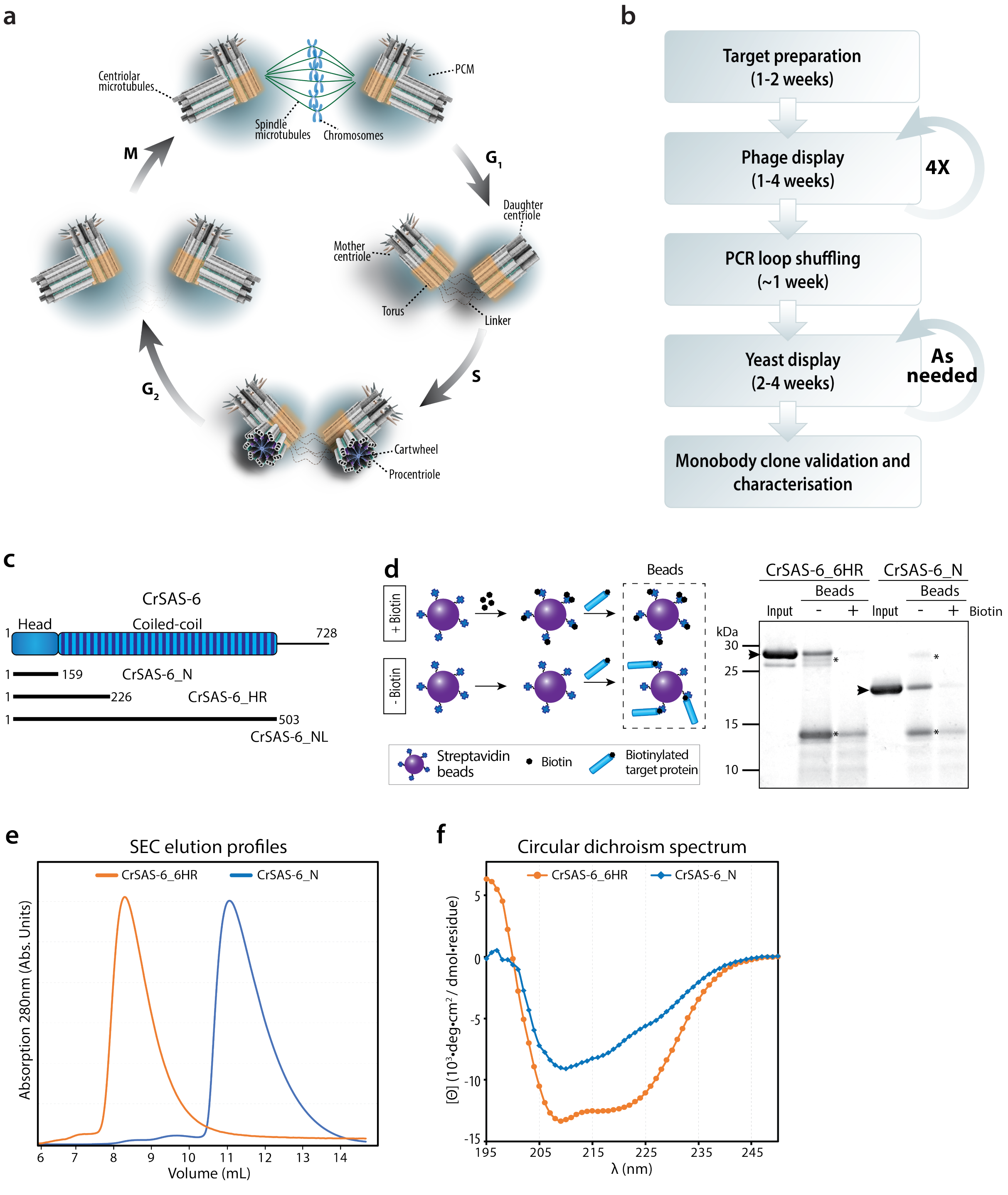

Supplementary figure 1: Centriole duplication cycle, monobody selection process and target validation prior to selection.

(**a**) Schematic of centriole duplication cycle. At the onset of the cell cycle (G_1), proliferating cells typically harbor two resident centrioles (mother and daughter centrioles) joined by a linker and surrounded by Peri-Centriolar Material (PCM); the appendages present on the mother centriole are also represented. Towards the G_1/S transition, one procentriole emerges from the surface of a torus surrounding the proximal part of both mother and daughter centriole. The cartwheel is the first structure apparent in the nascent procentriole; microtubules are then added to the forming organelle, which matures further thereafter. During mitosis, the two centriole/procentriole pairs, each surrounded by PCM, separate from one another, each serving as a spindle pole that promotes assembly of a bipolar spindle and faithful chromosome segregation.
(**b**) Flowchart of the monobody selection process with approximate duration of each step. See Methods for details.
(**c**) Schematic of the full length CrSAS-6 protein (top); the N-terminal globular head domain is in blue, the coiled-coil in striped blue, and the C-terminal moiety is shown as a black line. Protein fragments used in this study

(bottom); CrSAS-6_N and CrSAS-6_6HR are the targets used for monobody selection, whereas CrSAS-6_NL is used in the PORT-HS-AFM and cryo-EM experiments.
(**d**) Target biotinylation test; schematic on the left and SDS-PAGE on the right. Biotinylated CrSAS-6_N and CrSAS-6_6HR were immobilized on streptavidin coated beads. As a negative control, biotin blocked beads were used. The input and bound fractions were collected and subjected to SDS-PAGE. Arrows point to biotinylated CrSAS-6_N and CrSAS-6_6HR products, asterisks to bands corresponding to streptavidin degradation products. Molecular weight standards are indicated on the left in kDa. Single experiment.
(**e, f**) Elution profile from size exclusion chromatography (SEC) column (e) and circular dichroism spectrum (f) for CrSAS-6_N and CrSAS-6_6HR. Size exclusion chromatography data were collected using UNICORN 7.0 and plotted in Excel.

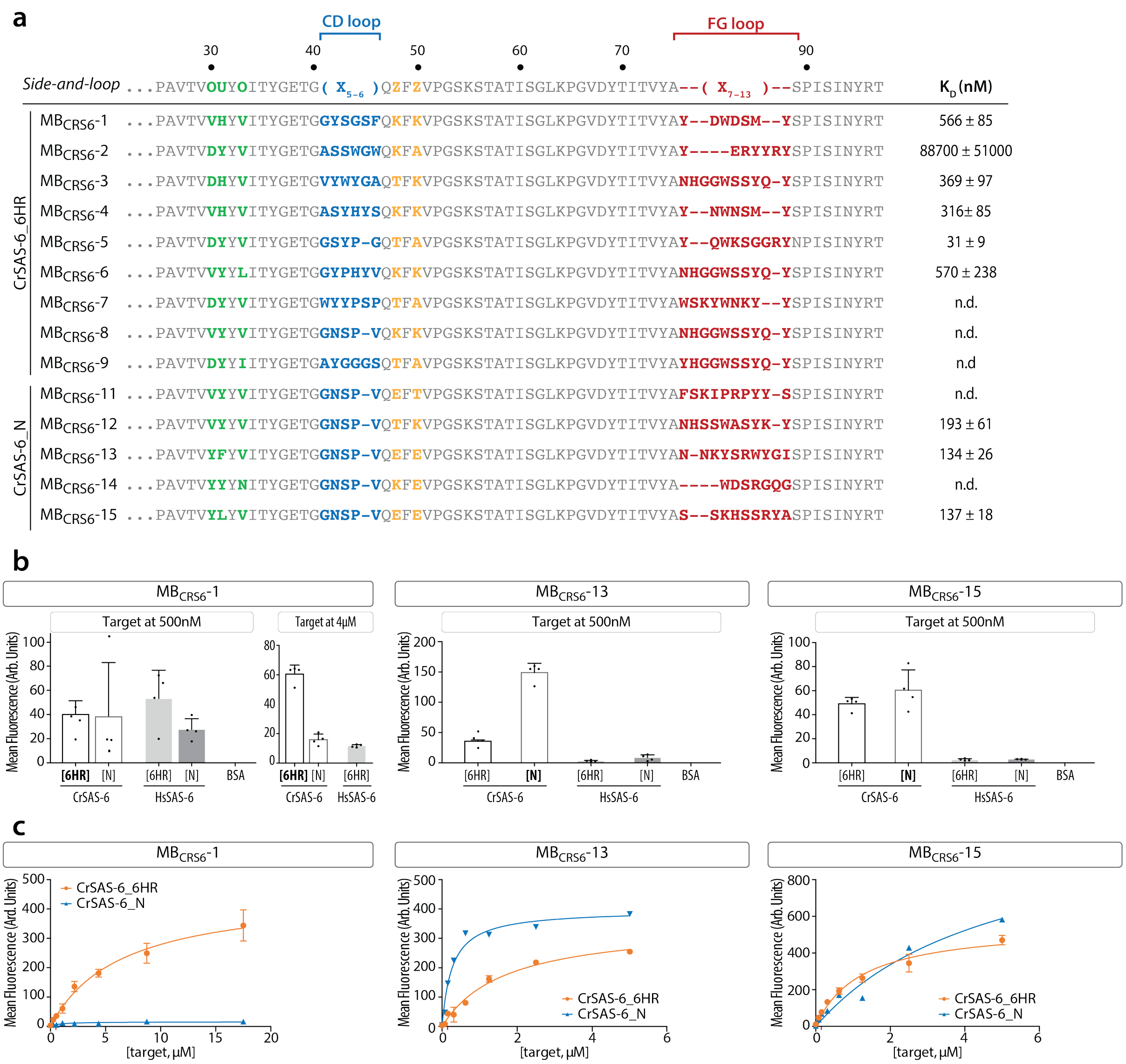

Supplementary figure 2: Outcome of selection process and monobody specificity test

(**a**) Sequence alignment of selected monobodies. MB_{CrS6}-1 to MB_{CrS6}-9 were selected against CrSAS-6_6HR, MB_{CrS6}-11 to MB_{CrS6}-15 against CrSAS-6_N. Residues in light gray are of the native monobody sequence. The variable residues in the library are colored: CD loop in blue, FG loop in red, side residues in the connecting ßC/ßD strands in green and yellow. Measured K_Ds, along with their standard deviation, are shown on the right.

(**b**) Yeast binding assay to determine binding specificity towards equivalent fragments of HsSAS-6 or a negative control (BSA) at fixed target concentration. Data represent mean ± SD from 4 replicates. The bold lettering indicates in each case the target against which the monobody was raised.

(**c**) Yeast binding assay at varying target concentrations of either the actual target or the other CrSAS-6 protein construct. Mean ± SD from two replicates for CrSAS-6_6HR titration, CrSAS-6_N titration single experiment.

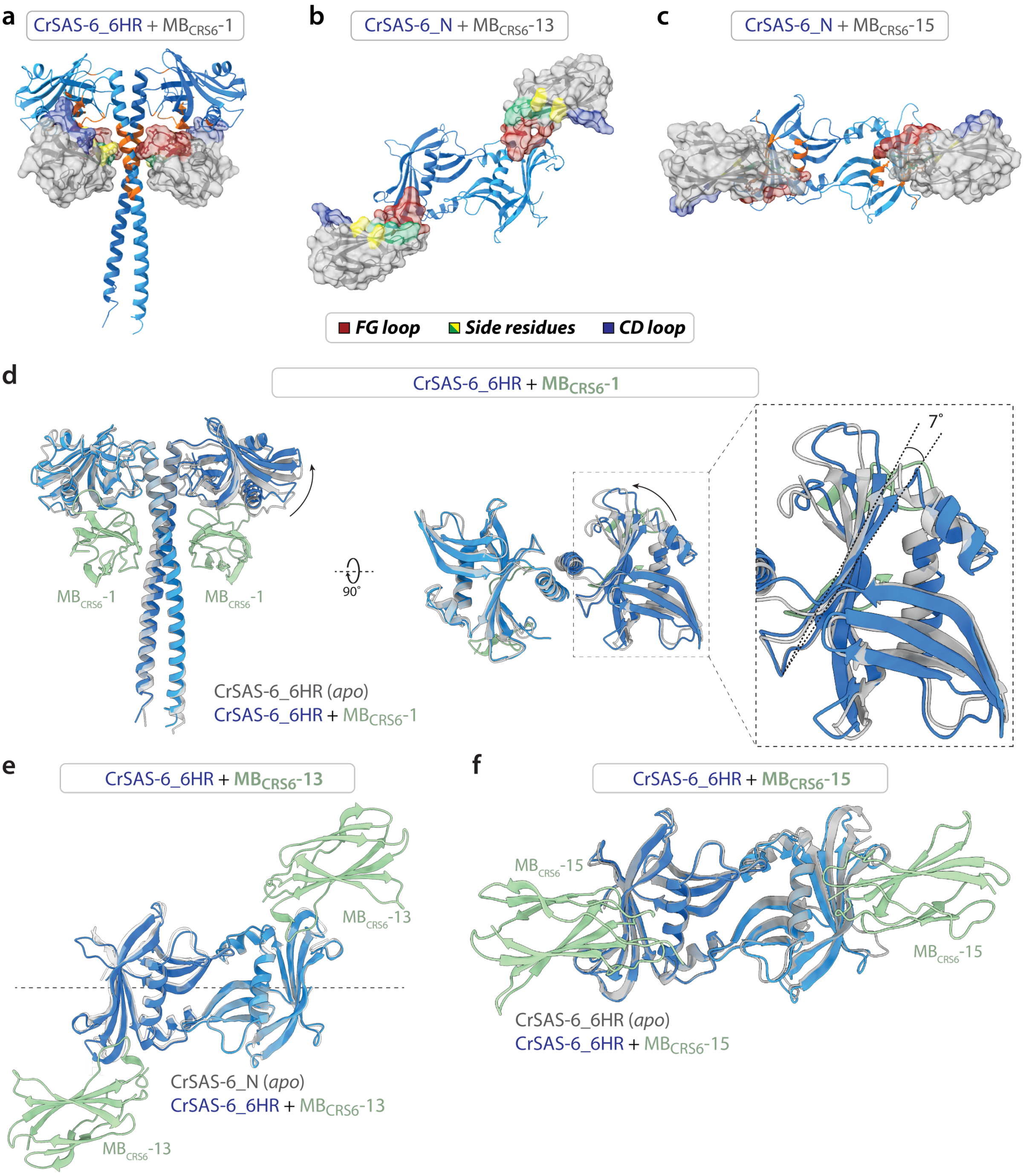

Supplementary figure 3: Monobody binding mode and structural comparisons of native CrSAS-6 with CrSAS-6 bound to monobodies

(**a-c**) Binding mode of monobodies MB_{CRS6}-1 (a), MB_{CRS6}-13 (b) and MB_{CRS6}-15 (c) to their respective target, highlighting the contribution of each type of diversified region, indicated in the same color code as in Fig. 1b.

(**d-f**) Comparison of CrSAS-6_6HR structure in native conformation (gray) with CrSAS-6_6HR in complex with MB_{CRS6}-1 (d), MB_{CRS6}-13 (e) or MB_{CRS6}-15 (f). CrSAS-6_6HR is shown in blue, monobodies in light green. Dotted line in (e) indicates the plane of the CrSAS-6 ring polymer.

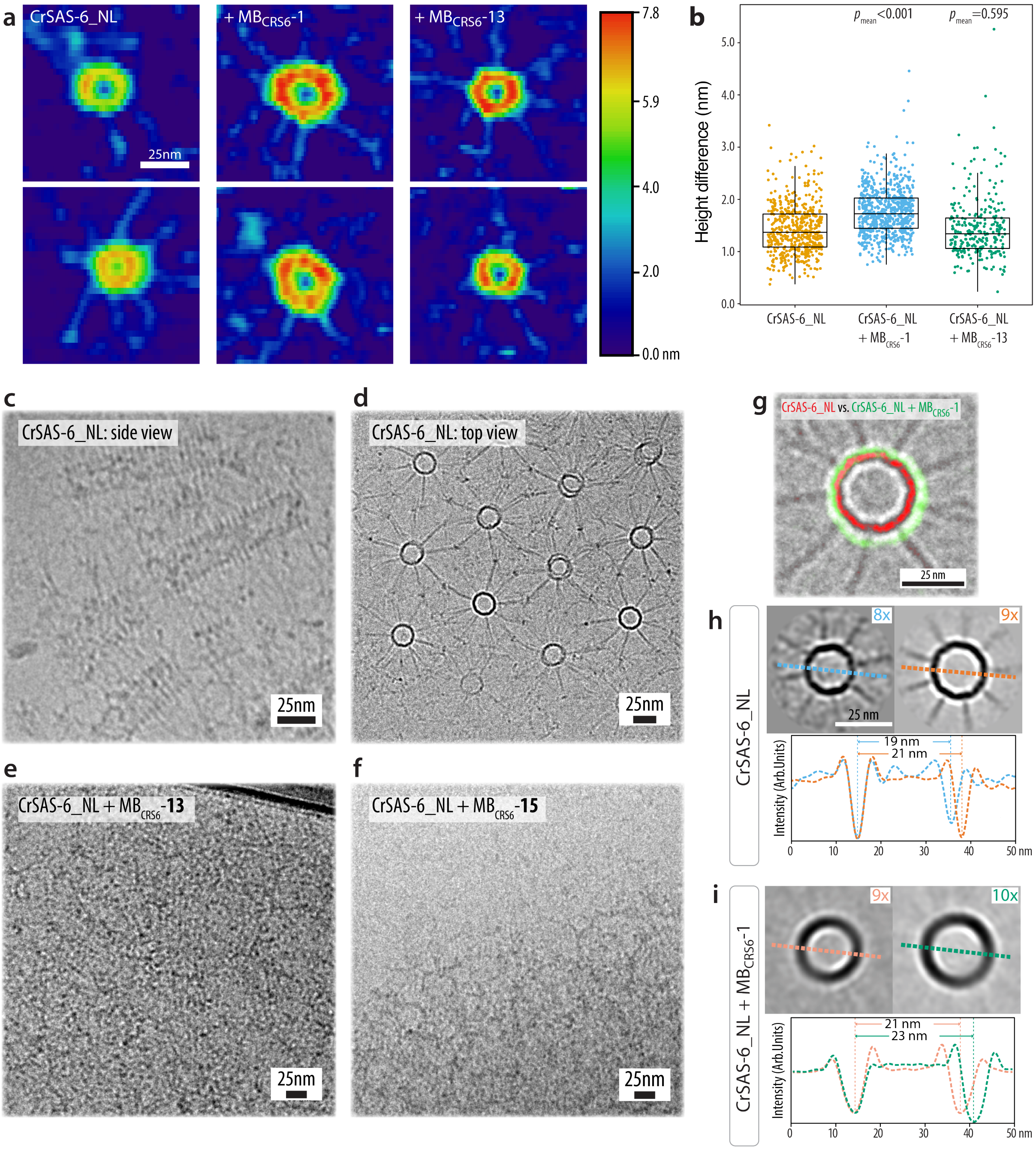

Supplementary figure 4: PORT-HS-AFM and cryo-EM analysis of monobody impact on CrSAS-6 ring assembly and stacking

(**a**) High magnification views of assemblies observed with PORT-HS-AFM equilibrium analysis of CrSAS-6_NL alone, and together with MB_{CRS6}-1 or MB_{CRS6}-13 (two representative examples of assemblies are shown in each case), highlighting height difference between the assembly types, as evident by examining the LUT on the right.

(**b**) Jittered boxplot of height difference (maximum minus minimum ring height, see Methods) observed using PORT-HS-AFM with CrSAS-6_NL alone, and with CrSAS-6_NL together with MB_{CRS6}-1 or MB_{CRS6}-13. Center lines show the means; box limits indicate the 25th and 75th percentiles; whiskers extend 1.5 times the interquartile range from the 25th and 75th percentiles. Number of rings analyzed: CrSAS-6_NL N=453, CrSAS-6_NL+MB_{CRS6}-1 N=530, and CrSAS-6_NL +MB_{CRS6}-13 N=272. The *p*-values were determined by a randomization test (CrSAS-6_NL versus CrSAS-6_NL+MB_{CRS6}-1 $p_{mean}<0.001$, CrSAS-6_NL versus CrSAS-6_NL+MB_{CRS6}-13 $p_{mean}>0.05$). Graphs and *p*-values were generated using PlotsOfDifferences[72].

(**c-f**) Cryo-EM side (c) and top (d) views of stacks of CrSAS-6_NL rings, as well as of CrSAS-6_NL with a 50% molar excess of MB_{CRS6}-13 (e) or of MB_{CRS6}-15 (f). Note that no ring stacks were observed in the presence of MB_{CRS6}-13 or M_{BCRS6}-15; note also that individual rings cannot be detected in this assay due to signal to noise limitations.

(**g**) Overlay of ring polymers from cryo-EM stacking assay with CrSAS-6_NL alone (red) or CrSAS-6_NL and a 50% molar excess of MB_{CRS6}-1 (green). Note that MB_{CRS6}-1 increases not only ring thickness but also inner ring diameter.
(**h, i**) 8-, 9- and 10-fold class averages, as indicated, of cryo-EM particles extracted from ring stacking assay with CrSAS-6_NL alone (h) or CrSAS-6_NL and a 50% molar excess of MB_{CRS6}-1 (i), with an indication regarding where ring symmetries were determined (dashed lines). Ring diameters for CrSAS-6_NL are 19 nm and 21 nm for 8- and 9- fold classes, respectively. For CrSAS-6_NL together with MB_{CRS6}-1 larger rings ~23 nm in diameter corresponding to 10- fold can also be observed (i, right). Note that the inner ring dimeter is expanded also in this case (13.7 nm versus 12.2 nm in the control condition).

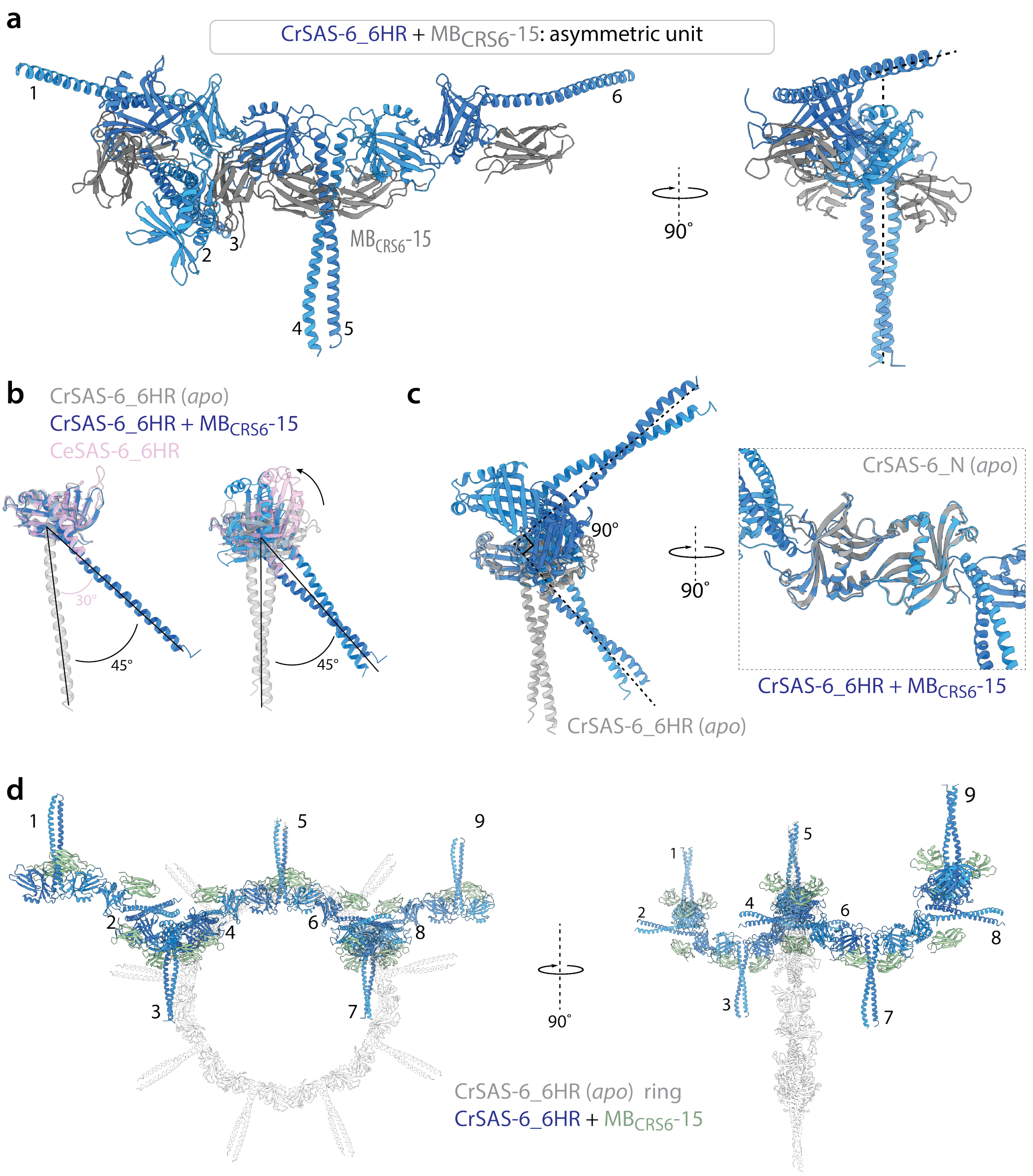

Supplementary Figure 5: MB_{CRS6}-15 induces helices in higher order CrSAS-6 oligomers

(**a**) Ribbon representation of the asymmetric unit (ASU) of CrSAS-6_6HR in complex with MB_{CRS6}-15. CrSAS-6_6HR molecules are shown in blue, monobodies in gray. There are six copies of the CrSAS-6_6HR—MB_{CRS6}-15 complex in the ASU.

(**b, c**) Superposition of N-terminal domains for SAS-6 proteins [gray: CrSAS-6_6HR alone (apo), blue: CrSAS-6_6HR in complex with MB_{CRS6}-15, pink: *C. elegans* SAS-6_6HR alone (apo, PDB ID: 4GFA)]. Note notably the extent of conformational change upon MB_{CrS6}-15 binding a single molecule of CrSAS-6_6HR (left) and on the homodimer (right). The coiled-coil is tilted by ~45° from its initial axis (b). The adjacent head domain is also tilted by ~45°, resulting to a total ~90° tilt between homodimers (c). Note that the head-to-head interface is similar to that observed in the crystal structure of CrSAS-6_N alone (apo). Note also that the tilt induced by MB_{CRS6}-15 resembles the tilt observed naturally in *C. elegans* SAS-6.

(**d**) Comparison of a 9-membered oligomer formed by CrSAS-6 in its native conformation (ring polymer, light gray, apo), and upon the conformational change imparted by MB_{CRS6}-15 (light green) binding (helical polymer, blue).

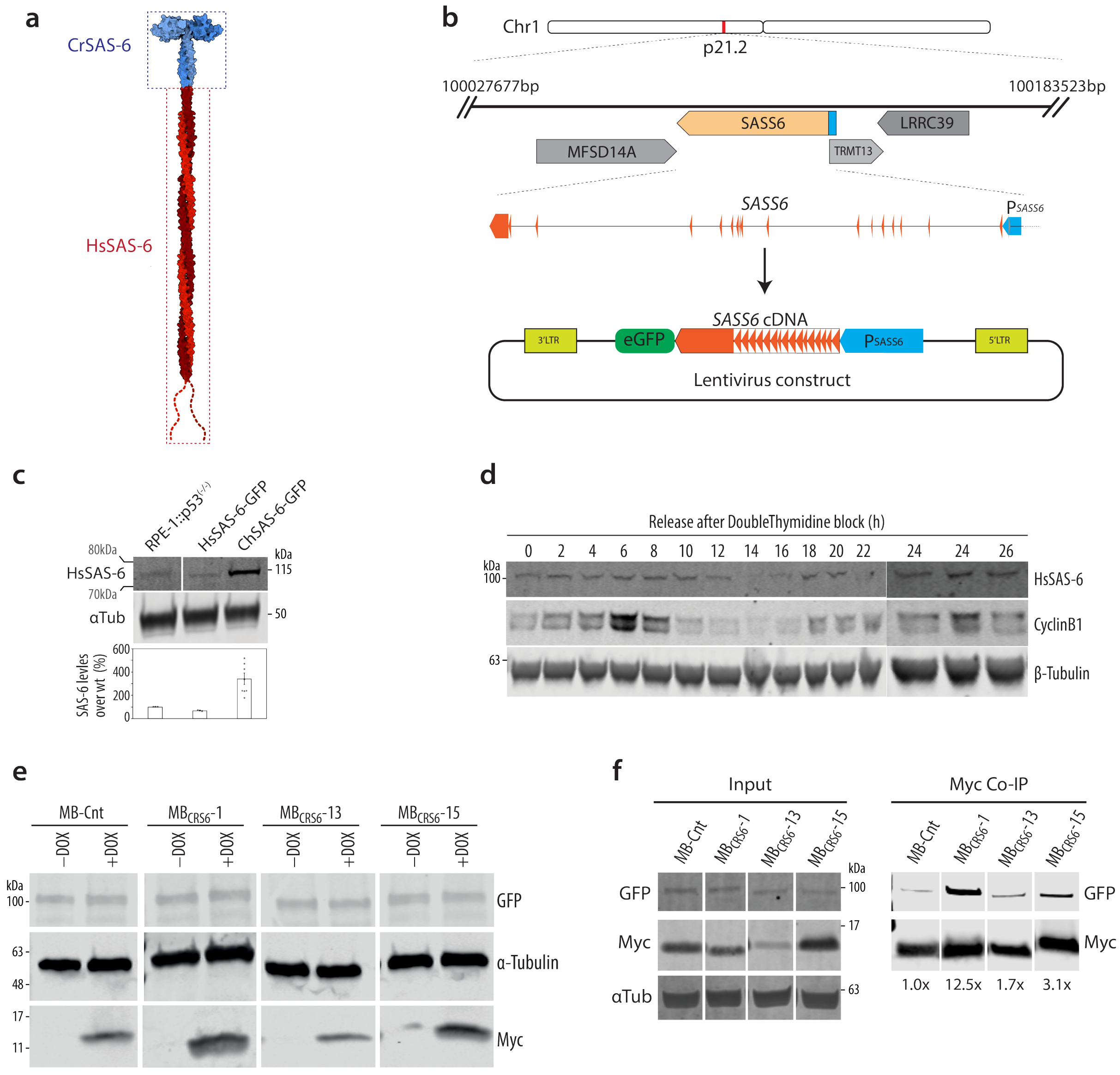

Supplementary Figure 6: **Generation of chimeric SAS-6 construct and cell lines for *in cellulo* experiments**
(**a**) Schematic of chimeric SAS-6 protein, with the N-terminal region from CrSAS-6 (amino acids 1-204, blue) fused to the coiled-coil and C-terminus domains from HsSAS-6 (amino acids 191-657, red).
(**b**) Schematic of mini-HsSAS-6-GFP construct, whereby the *SASS6* cDNA was fused to eGFP and driven from the native *sass6* promoter.
(**c**) Western blot of lysates from indicated cell lines probed with antibodies against HsSAS-6 (top) or α-tubulin as loading control (bottom), with corresponding quantification from 3 independent experiments (mean ± SEM).
(**d**) Western blot of lysates from cells expressing HsSAS-6-eGFP collected at indicated times following release from a double thymidine block; the blots were probed with antibodies against GFP to monitor HsSAS-6-eGFP (top), Cyclin B1 to monitor cell cycle progression (middle) and β-tubulin as loading control (bottom).
(**e**) Doxycycline addition triggers expression of the monobodies tagged with Myc.
(**f**) Co-immunoprecipitation of ChSAS-6-GFP by monobodies tagged with Myc, with corresponding quantification of ChSAS-6 normalized against the control lane, single experiment.

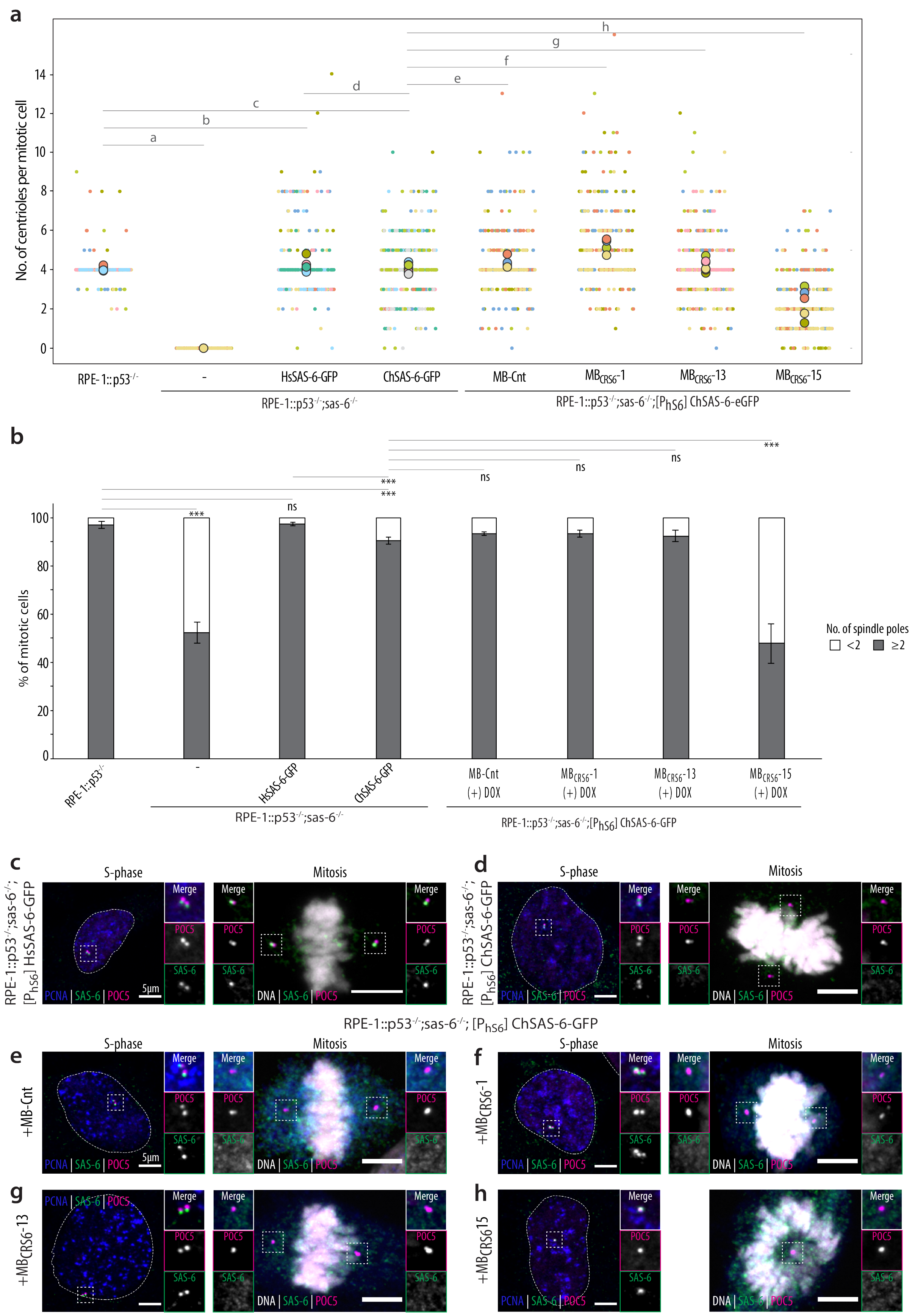

Supplementary Figure 7: Supporting information for *in cellulo* experiments
(**a**) Detailed quantification of centriole number from experiments shown in Figure 4e and 4j. The data is shown as a SuperPlot, with colors representing different replicates. Large circles mark the median for each replicate. The *p*-

values were determined by a randomization test (a: *p_median* < 0.001, b: *p_median* = 1, c: *p_median* = 1, d: *p_median* = 1, e: *p_median* = 1, f: *p_median* < 0.001, g: *p_median* = 1, h: *p_median* < 0.001). Graphs and *p*-values are generated using SuperplotsOfData and SuperplotsOfDifferences[1-3].

(**b**) Quantification of mitotic cells with <2 or ≥2 spindle poles. Data represent mean ± SEM. Number of cells analyzed in at least 3 independent experiments: RPE-1::p53$^{-/-}$ N=406, RPE-1::p53$^{-/-}$;sas-6$^{-/-}$ N=275, RPE-1::p53$^{-/-}$;sas-6$^{-/-}$ + HsSAS-6-GFP N=527, RPE-1::p53$^{-/-}$;sas-6$^{-/-}$ + ChSAS-6-GFP N=634, RPE-1::p53$^{-/-}$;sas-6$^{-/-}$ + ChSAS-6-GFP + MB-Cnt N=326, RPE-1::p53$^{-/-}$;sas-6$^{-/-}$ + ChSAS-6-GFP + MB$_{CRS6}$-1 N=270, RPE-1::p53$^{-/-}$;sas-6$^{-/-}$ + ChSAS-6-GFP + MB$_{CRS6}$-13 N=325, RPE-1::p53$^{-/-}$;sas-6$^{-/-}$ + ChSAS-6-GFP+MB$_{CRS6}$-15 N=273. Statistical analysis was performed using Yate's chi-square test (RPE-1::p53$^{-/-}$ versus RPE-1::p53$^{-/-}$;sas-6$^{-/-}$ *p*<0.001; RPE-1::p53$^{-/-}$ versus RPE-1::p53$^{-/-}$;sas-6$^{-/-}$ + HsSAS-6-GFP *p*=0.880; RPE-1::p53$^{-/-}$ versus RPE-1::p53$^{-/-}$;sas-6$^{-/-}$ + ChSAS-6-GFP *p*<0.001; RPE-1::p53$^{-/-}$;sas-6$^{-/-}$ + HsSAS-6-GFP versus RPE-1::p53$^{-/-}$;sas-6$^{-/-}$ + ChSAS-6-GFP *p*<0.001; RPE-1::p53$^{-/-}$;sas-6$^{-/-}$ + ChSAS-6-GFP versus RPE-1::p53$^{-/-}$;sas-6$^{-/-}$ + ChSAS-6-GFP + MB-Cnt *p*=0.235, RPE-1::p53$^{-/-}$;sas-6$^{-/-}$ + ChSAS-6-GFP+MB$_{CRS6}$-1 *p*=0.503, and RPE-1::p53$^{-/-}$;sas-6$^{-/-}$ + ChSAS-6-GFP+MB$_{CRS6}$-13 *p*=0.437; RPE-1::p53$^{-/-}$;sas-6$^{-/-}$ + ChSAS-6-GFP versus RPE-1::p53$^{-/-}$;sas-6$^{-/-}$ + ChSAS-6-GFP + MB$_{CRS6}$-15 *p*<0.001). *** $p < 0.001$, ** $p < 0.01$, * $p < 0.05$, ns $p \geq 0.05$.

(**c-h**) Confocal images of RPE-1::p53$^{-/-}$;sas-6$^{-/-}$ cells in S-phase (left columns) or mitosis (right columns) expressing HsSAS-6-GFP (c) or ChSAS-6-GFP (d-h), as well as the indicated monobodies in addition (e-h). Cells were stained with antibodies against the Centrin-binding protein POC5 (to mark centrioles, magenta), HsSAS-6 (to detect HsSAS-6 and ChSAS-6, green) and PCNA (to detect cells in S phase, blue), as well as counterstained with a DNA dye (gray). Scale bars: 5µm. Two experimental replicates were performed.

References

1. Goedhart, J. PlotsOfDifferences - a web app for the quantitative comparison of unpaired data. *bioRxiv* 578575 (2019). doi:10.1101/578575
2. Goedhart, J. SuperPlotsOfData – a web app for the transparent display and quantitative comparison of continuous data from different conditions. *Mol. Biol. Cell* mbc.E20-09-0583 (2021). doi:10.1091/mbc.e20-09-0583
3. Lord, S. J., Velle, K. B., Dyche Mullins, R. & Fritz-Laylin, L. K. SuperPlots: Communicating reproducibility and variability in cell biology. *Journal of Cell Biology* **219**, (2020).

Monobody	Sample in cell	[Cell] (µM)	Sample in syringe	[Syringe] (µM)	State	KD (nM)	KD Error (µM)	N (sites)	N Error (sites)	ΔH (kcal/mol)	ΔH Error (kcal/mol)	ΔG (kcal/mol)	-TΔS (kcal/mol)	Offset (kcal/mol)	Offset Error (kcal/mol)	Red. Chi-Sqr. (kcal/mol)²
MB_{CRS6}-1	CrSAS-6_6HR	12	MB_{CRS6}-1	150	Binding	566.0	85.1	0.937	0.014	-15.40	0.42	-8.52	6.87	0.008	0.168	0.084
MB_{CRS6}-1	MB_{CRS6}-1	20	CrSAS-6_N	200	No binding											
MB_{CRS6}-2	CrSAS-6_6HR	12	MB_{CRS6}-2	150	Binding	88'700.0	51'100.0	1.000	0.000	-123.00	79.90	-5.53	118.00	1.780	2.230	0.056
MB_{CRS6}-3	CrSAS-6_6HR	12	MB_{CRS6}-3	150	Binding	369.0	97.0	0.790	0.019	-18.60	0.78	-8.78	9.84	-0.928	0.280	0.365
MB_{CRS6}-4	MB_{CRS6}-4	12	CrSAS-6_6HR	150	Binding	316.0	84.9	0.810	0.019	-14.50	0.58	-8.87	5.65	-0.385	0.222	0.238
MB_{CRS6}-5	CrSAS-6_6HR	20	MB_{CRS6}-5	200	Binding	31.1	8.7	0.893	0.006	-10.50	0.17	-10.20	0.28	0.301	0.114	0.055
MB_{CRS6}-6	CrSAS-6_6HR	10	MB_{CRS6}-6	122	Binding	570.0	238.0	0.720	0.036	-23.90	2.13	-8.52	15.40	-12.200	0.613	1.680
MB_{CRS6}-12	CrSAS-6_6HR	9	MB_{CRS6}-12	180	Binding	193.0	60.9	1.760	0.031	-2.28	0.09	-9.16	-6.88	-0.270	0.046	0.011
MB_{CRS6}-13	CrSAS-6_N	15	MB_{CRS6}-13	146	Binding	134.0	26.0	0.766	0.008	-28.60	0.59	-9.38	19.20	-17.600	0.307	0.609
MB_{CRS6}-15	CrSAS-6_6HR	30	MB_{CRS6}-15	300	Binding	468.0	200.0	1.260	0.032	-2.49	0.17	-8.64	-6.14	-1.080	0.119	0.018
MB_{CRS6}-15	CrSAS-6_N	16	MB_{CRS6}-15	160	Binding	137.0	18.3	0.959	0.006	-12.50	0.18	-9.36	3.10	-0.353	0.111	0.060

Supplementary table 1: Details on ITC measurements.
Protein concentrations used are reported for both the sample in the cell and in the syringe, along with the results of the fit using a one site binding model. We report binding constant (K_D), reaction stoichiometry (N), enthalpy (ΔH), Gibbs free energy (ΔG), entropy (-TΔS), along with values for the quality of the fit.

Protein purification (vector / construct)	Primers (5'→3')
pHFT / CrSAS-6_6HR	GCCCGCTTCTTCTCGACGACGGTG
	GCCTCGAGTCACGTCTTCGCCTGGGCCTG
pHFT / CrSAS-6_N	GCCCGCTTCTTCTCGACGACGGTG
	GCCTCGAGTCAGTTGCCGGGCCGGAAGG
pFLOAT2-His / CrSAS-6_NL	TCTGTTCCAGGGGCCCATGCCGCTTCTTCTCGACG
	TTAGCAGCCGGATCTCTCAGGTCGCGCCTGAC
pHFT / Monobody	CGGGATCCGTTTCTTCTGTTCCGACCAAAC
	CGCTCGAGCTAGGTACGGTAGTTAATCGAGATTG
pET30a / ChSAS-6	GCGGTACCATGCCGCTTCTTCTCGAC
	AGAGCTCCTGCCGGCACTGCGC
	CGAGCTCGATAAGTTACGGAA
	CGAGCTCTTAACTGTTTGGTAACTG
In cellulo experiments (vector/construct)	Primers (5'→3')
pENTR1A-eGFP / ChSAS-6_F	GCactagtaccATGCCGCTTCTTCTCG
	GCactagtACTGTTTGGTAACTGCCCAGGG
pENTR1A-eGFP / HsSAS-6_F	GCactagtaccATGCCGCTTCTTCTCGacgac
	GCactagtACTGTTTGGTAACTGCCCAGGG
hPGK-GW-IRES-GFP / HsSAS-6	CGCTCGAGATGAGCCAAGTGCTGTTCCAC
	CGGCTAGCTTACTTGTACAGCTCGTCCATGCC
	CGCTCGAGGTTGGCTCGCTGCCTCG
	GTCTCGAGCTCCTGACCTCAAGTG
hPGK-GW-IRES-GFP / ChSAS-6	CGCTCGAGATGCCGCTTCTTCTCGACG
	CGGCTAGCTTACTTGTACAGCTCGTCCATGCC
pENTR1A-Myc / Monobody	CGGGATCCACCATGGTTTCTTCTGTTCCGACCAAACT
	GCGGATCCGGTACGGTAGTTAATCGAGATTGG

Supplementary Table 2: Constructs and primer list.
