# Supplementary material for: Tuning SAS-6 architecture with monobodies impairs distinct steps of centriole assembly
Source: Nat Commun. 2021 Jun 21;12:3805. doi: 10.1038/s41467-021-23897-0 (PMC8217511; doi:10.1038/s41467-021-23897-0)
Supplement: Supplementary file 3 — Description of Additional Supplementary Files [file 41467_2021_23897_MOESM3_ESM.docx]

**Description of Additional Supplementary Files**

File Name: **Supplementary Movie 1: PORT-HS-AFM uncovers impact of monobodies on CrSAS-6 self-assembly**

Description: PORT-HS-AFM of CrSAS-6_NL self-assembly reactions in the absence (left, from^26^) or presence of MBCrS6-15 added at the onset of the assembly reaction (right).

File Name: **Supplementary Movie 2: Conformational change imposed on CrSAS-6_6HR upon MBCRS6-15 binding**

Description: CrSAS-6_6HR is shown in its native conformation (two shades of blue) and in a complex with MBCRS6-15 (grey). MBCRS6-15 induces a large conformational change, resulting in a -45° rotation between the head domain and the coiled-coil moiety.

File Name: **Supplementary Movie 3: Ring to helix transition upon MBCRS6-15 binding**

Description: CrSAS-6_6HR ring is shown in blue at first and in light grey thereafter. MBCRS6-15 induces a large conformational alteration, resulting in a -90° rotation between adjacent homodimers, leading to the formation of a helix with a 4-fold screw axis.
