# Supplementary material for: Tuning SAS-6 architecture with monobodies impairs distinct steps of centriole assembly
Source: Nat Commun. 2021 Jun 21;12:3805. doi: 10.1038/s41467-021-23897-0 (PMC8217511; doi:10.1038/s41467-021-23897-0)

Supplementary source data - Gels & immunoblots

Related to Supplementary figure 1d - Uncropped

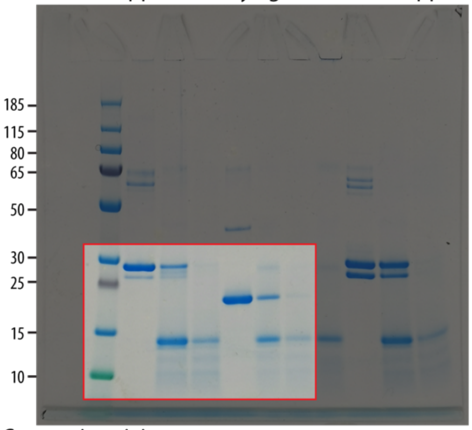

Coomassie staining

Related to Supplementary figure 6c - Uncropped

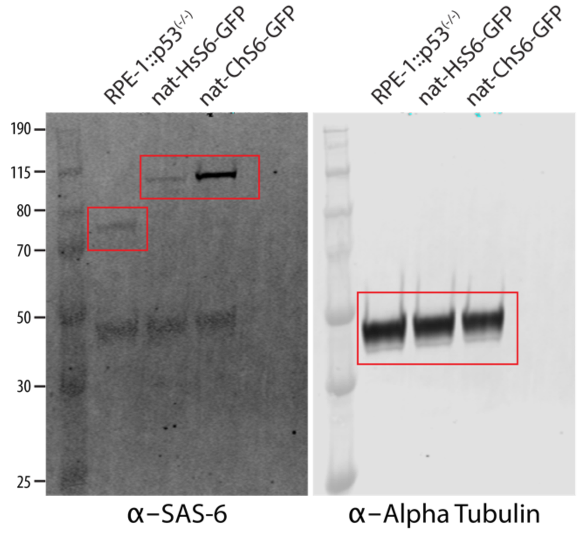

Related to Supplementary figure 6d - Uncropped

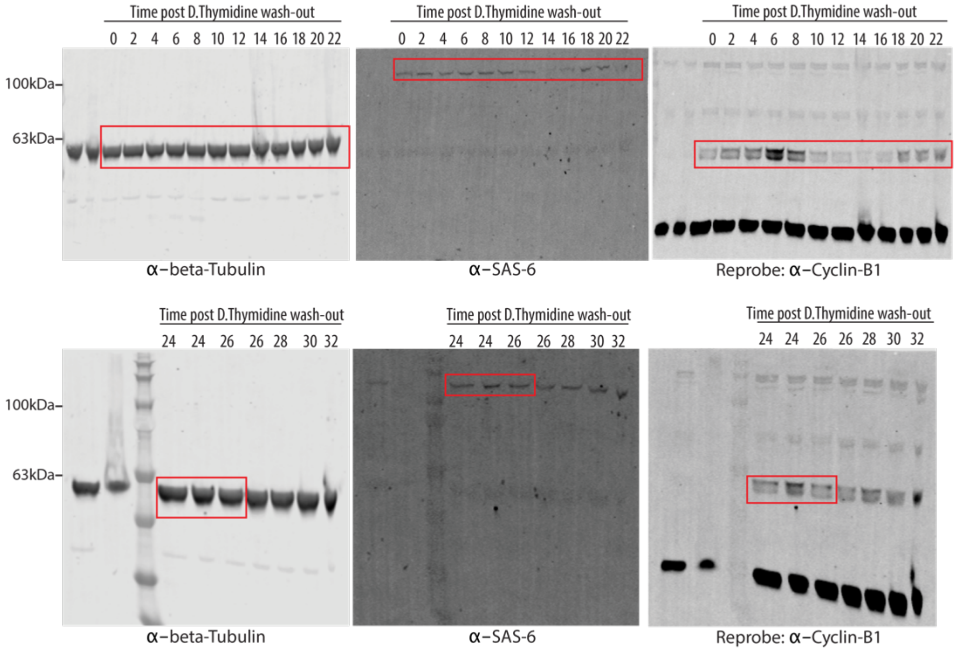

Related to Supplementary figure 6e - Uncropped

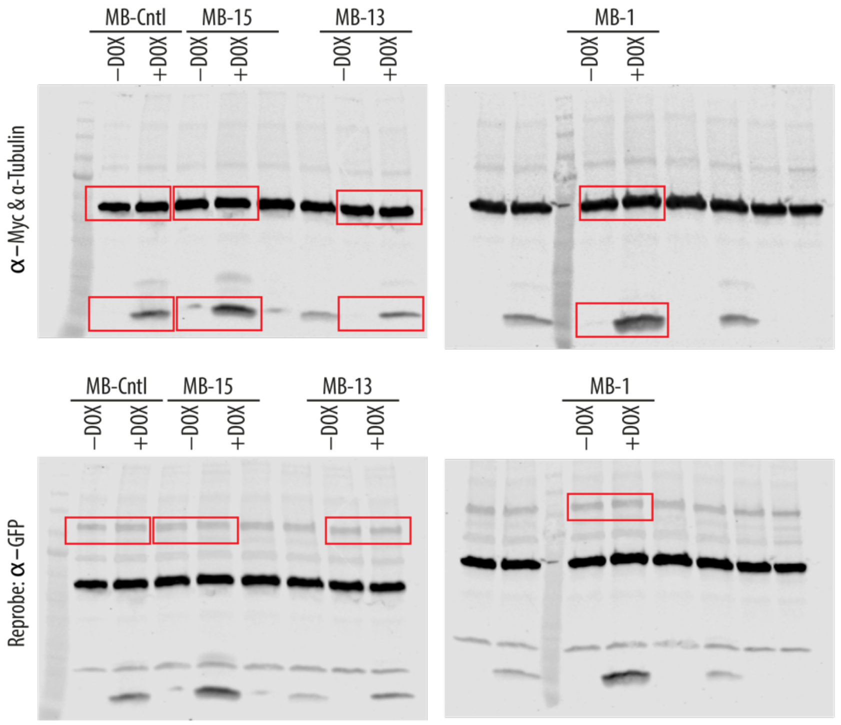

Related to Supplementary figure 6f - Uncropped

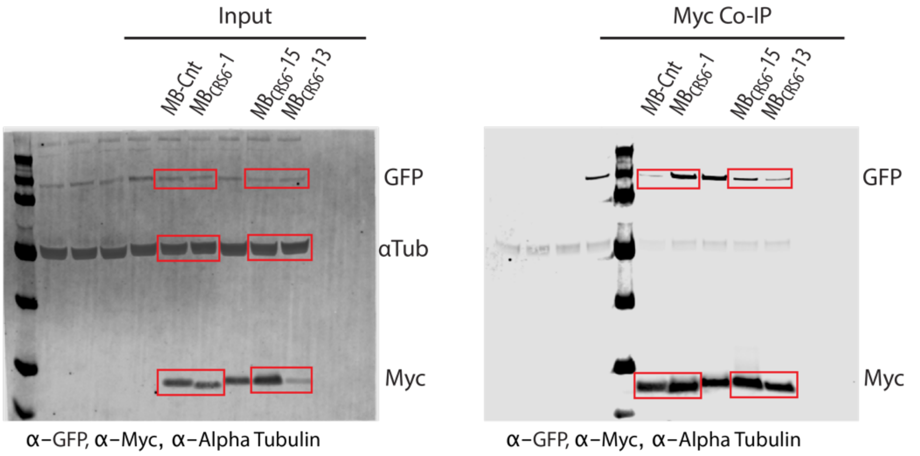

Supplement: Supplementary file 7 — Source Data [file 41467_2021_23897_MOESM7_ESM.zip › Soource Data-Gels_Blots.pdf]
