# Supplementary material for: Tuning SAS-6 architecture with monobodies impairs distinct steps of centriole assembly
Source: Nat Commun. 2021 Jun 21;12:3805. doi: 10.1038/s41467-021-23897-0 (PMC8217511; doi:10.1038/s41467-021-23897-0)
Supplement: Supplementary file 2 — Peer Review File [file 41467_2021_23897_MOESM2_ESM.pdf]

Reviewer #2 (Remarks to the Author):

I believe that the authors have sufficiently addressed my concerns to the previous manuscript in this revised version. Therefore, I recommend this manuscript to publish in Nature Communication.

Reviewer #3 (Remarks to the Author):

In this work, the authors have identified polypeptide-based inhibitors, monobodies that can interfere centriole cartwheel formation and thereby, centriole duplication. They have used multiple biophysical techniques to validate the inhibitory potentials of the monobody and have shown its effectiveness in cell. The results are of high importance and they provide a novel strategy in targeting centrosome.
Authors have addressed most of my concerns with reasonable explanations and additional data. I recommend acceptance of the paper.

Reviewer #4 (Remarks to the Author):

The issues identified in the initial round of reviews have been addressed by the authors and no additional revision is required.
